# A SAM2 and VLM-Based Automatic Radiological Reporting System for Pneumothorax Detection

**DOI:** 10.3390/biomedicines14102312

**Published:** 2026-10-12

**Authors:** Savaş Tunçer, Mustafa Yıldırım, Özal Yıldırım

**Affiliations:** 1 Faculty of Technology, Department of Software Engineering Firat University, Elazig 23000, Türkiye; savastuncer62@gmail.com; 2 Faculty of Medicine Hospital, Department of Radiology, Firat University, Elazig 23000, Türkiye; m.yildirim@firat.edu.tr

**Keywords:** pneumothorax, Segment Anything Model 2, vision-language model, U-Net, medical image segmentation, automatic radiological reporting

## Abstract

**Background/Objectives:** Pneumothorax, caused by air accumulation in the pleural space, can lead to lung collapse and, if diagnosis is delayed, respiratory or cardiovascular deterioration. Most deep-learning studies on pneumothorax address classification or segmentation alone. This study proposes a holistic automatic radiological reporting system combining U-Net-based segmentation, Segment Anything Model 2 (SAM2)-based boundary refinement, and a Vision-Language Model (VLM)-based reporting module for pneumothorax detection on computed tomography (CT) images. **Methods:** A MobileNetV2-encoder U-Net was trained on 224×224 CT patches from 171 patients (15,555 patches). A frozen, zero-shot SAM2 module, guided by a box prompt derived from the U-Net mask, was combined with U-Net via an Intersection over Union (IoU)-agreement rule. The refined mask and rule-based geometric features were passed to a frozen VLM (Qwen2-VL-2B-Instruct) to generate structured reports with a combined confidence coefficient. Performance was evaluated with Accuracy, Dice, IoU, and Loss, over all patches and over pneumothorax-positive patches only. **Results:** U-Net achieved 93.09/92.09/99.71% (Dice/IoU/Accuracy) on validation and 93.68/92.79/99.79% on the test set; these largely reflect correct empty-mask prediction on negative patches. On positive patches only, Dice was 0.6295 (validation) and 0.6215 (test), with patch-level sensitivity of 82.2% (validation) and 79.9% (test) and specificity above 97%. SAM2 refinement achieved 92.44% Dice and 91.29% IoU on a random test subsample (*n* ≈ 300), close to but not exceeding U-Net alone; an ablation analysis confirmed no consistent Dice/IoU or 95th-percentile Hausdorff distance (HD95) gain from SAM2. BLEU/ROUGE and Likert evaluation frameworks for the VLM reports were defined but not yet scored against reference reports. **Conclusions:** U-Net-based segmentation reliably identifies pneumothorax-containing patches with moderate boundary accuracy, while frozen SAM2 refinement adds limited value. The rule-based reporting layer is reproducible and shows triage-support potential, whereas the VLM free-text layer carries a documented hallucination risk and requires radiologist verification before clinical use.

## 1. Introduction

Pneumothorax is defined as the partial or complete collapse of the lung due to an abnormal accumulation of air in the pleural space [1]. The condition is typically characterized by sudden chest pain, shortness of breath, and impaired respiratory function. Early diagnosis is of critical importance, particularly in emergency departments and intensive care units. Delays in diagnosis can adversely affect the course of the disease, leading to severe respiratory distress and potentially life-threatening conditions [2].

Chest radiography (chest X-ray) and computed tomography (CT) are widely used today in the diagnosis of pneumothorax. Chest radiography is the first-line imaging modality for emergency assessment owing to its low cost, rapid accessibility, and suitability for bedside application. Computed tomography provides high accuracy but has limitations such as high cost, radiation exposure, and the need for expert evaluation [3]. The rapidly increasing number of imaging studies in radiology departments increases the workload of specialists and can lead to delays in the evaluation process. Deep-learning-based detection algorithms have therefore been investigated as a means of supporting radiological workflows, including the surveillance of pneumothorax in routine clinical cohorts [4]. The images used in this study consist of computed tomography slices. Figure 1 shows example computed tomography (CT) slices containing pneumothorax and their corresponding reference annotations.

In recent years, advances in artificial intelligence and deep-learning-based methods have led to significant progress in medical image analysis [5,6]. Convolutional Neural Network (CNN)-based models have demonstrated high accuracy rates on medical images [7]. In medical artificial intelligence applications, explainability methods such as Grad-CAM [8] and SHAP [9] have emerged as tools supporting decision mechanisms, aiming to clinically justify model decisions. Large-scale, openly accessible chest radiography datasets and benchmark studies supporting these advances have accelerated the development of automatic detection systems. In this regard, datasets widely used in the literature, such as MIMIC-CXR [10] and CheXpert [11], along with reference models such as CheXNet [12], have laid the groundwork for clinical studies. In particular, the U-Net architecture developed by Ronneberger et al. has become one of the fundamental methods in biomedical image segmentation [13]. Owing to its encoder–decoder structure and skip connections, U-Net has produced successful results in delineating small anatomical structures. In subsequent years, performance improvements have been achieved with U-Net++, Attention U-Net, and transformer-based models [14]. In such network architectures, deep convolutional backbones such as ResNet [15], DenseNet [16], Inception [17], and EfficientNet [18] are frequently preferred as the encoder component.

One of the major developments in the field of image segmentation has been the Segment Anything Model (SAM) developed by Meta and its improved version, Segment Anything Model 2 (SAM2) [19,20]. Owing to its large-scale pretraining process, SAM2 can delineate the boundaries of diverse objects with high accuracy. It has been shown to achieve strong performance on medical images containing complex anatomical structures [21]. In the optimization of deep networks for medical imaging, adaptive gradient-based optimization algorithms such as Adam [22] directly affect convergence speed and accuracy. The studies of Litjens et al. [7] and Esteva et al. [23] demonstrate the generalization capability of deep learning in medical diagnostic processes and its success in clinical classification.

However, the vast majority of existing studies focus solely on the segmentation task. Yet, in clinical practice, identifying the pathological region alone is not sufficient; interpretation of the resulting images by expert radiologists is equally important. Vision-Language Model (VLM) systems can generate automatic clinical interpretations by combining visual data with natural language processing models [24]. This approach enables image analysis and clinical interpretation to be carried out within the same pipeline.

In this study, U-Net-based initial segmentation, SAM2-based segmentation refinement, and VLM-based structured reporting are brought together for pneumothorax detection. The proposed system aims not only to identify pneumothorax regions but also to convert the segmentation outputs into explainable clinical interpretations.

The remainder of this section reviews related work on segmentation and automatic reporting methods and identifies the gaps that motivate this study; Section 2 describes the dataset and the proposed methods; Section 3 presents the experimental results; Section 4 discusses the findings, practical implications, and limitations; and Section 5 concludes the paper.

Deep-learning-based methods have achieved significant progress in medical image analysis and have increasingly been used, in particular, for the automatic detection of chest diseases. Dependence on expert opinion, variability in interpretation time, and observer-dependent variability constitute important limitations in traditional imaging assessments. Deep-learning-based detection systems have been proposed to reduce this dependence and have been evaluated in multicentre clinical cohorts for pneumothorax detection [4]. For this reason, automatic decision-support systems have become one of the important research areas.

One of the widely used methods in biomedical image segmentation is the U-Net architecture. U-Net, proposed by Ronneberger et al. [13], can achieve high accuracy even on limited datasets owing to its encoder–decoder structure. The encoder layers extract multi-level features from the image, while the decoder part reconstructs the image. Skip connections help preserve low-level spatial features. In subsequent years, improved models such as Attention U-Net, U-Net++, and DeepLabv3+ have been proposed and have improved performance [14,25,26,27]. In these models, architectures such as ResNet [15], DenseNet [16], Inception [17], EfficientNet [18], and Xception [28] have frequently been used as the encoder component.

In studies on pneumothorax detection, deep learning methods are seen to be widely used, particularly on chest radiographs and computed tomography slices. Abedalla et al. [29] performed pneumothorax segmentation on the SIIM-ACR chest radiography dataset using ensembles of U-Net with EfficientNet and ResNet encoders and obtained a high Dice coefficient. Similarly, Sae-Lim et al. [30] developed a two-stage deep learning system for automatic pneumothorax segmentation and quantitative assessment from chest radiographs. Although these studies produced good segmentation performance, they focused solely on identifying the disease region.

New approaches have emerged with the adoption of the transformer architecture in image processing. The attention mechanism proposed by Vaswani et al. [31] laid the foundation for these developments, and the Vision Transformer (ViT) model developed by Dosovitskiy et al. [32] demonstrated that the attention mechanism could be applied to image processing. Subsequently, the Segment Anything Model (SAM), developed by Kirillov et al. [19], was introduced into the literature as a foundation segmentation model trained on large-scale datasets.

SAM2, developed by Meta, is an improved version of the SAM model and can perform segmentation on both image and video data [20]. Owing to its multi-scale feature extraction and powerful encoder structure, it can delineate complex object boundaries. Recent studies have shown that SAM2-based systems can achieve high performance in medical imaging [21].

Alongside advances in image processing, progress in natural language processing models has also led to the adoption of Vision-Language Model (VLM)-based systems in medical image analysis. VLM architectures can generate descriptive text from images by combining visual features with language models [24]. Jing et al. [33] combined image features with natural language models for automatic report generation from medical images. Boecking et al. [34] showed that biomedical vision-language pretraining can improve reporting performance. More recently, general-purpose vision-language models combined with large language models (e.g., Qwen2-VL [35]) and their medically adapted variants (e.g., LLaVA-Med [36]) represent a new approach to free-text generation from images. In addition, explainability methods such as Grad-CAM [8] and SHAP [9] stand out as tools supporting the clinical interpretation of model decisions.

Beyond individual system-level studies, broader surveys have also mapped the trajectory of vision-language modeling in medical image analysis, from early feature-fusion approaches to general-purpose large multimodal models, and have highlighted report generation and grounded clinical interpretation as an underserved application area relative to classification and segmentation [37]. In parallel, knowledge-distillation and teacher–student strategies have been reviewed as a route to obtaining compact, deployable models for resource-constrained clinical settings without a large loss in accuracy [38]; such strategies are directly relevant to the lightweight-encoder design choice adopted in Section 2.3 and to the future deployment of the VLM component of the present system.

Table 1 summarizes representative studies discussed above, together with their imaging modality, method, principal reported result, and the gap that each leaves relative to the system proposed in this paper.

As summarized in Table 1, a review of the literature shows that the vast majority of existing studies focus solely on image classification or segmentation. Studies that jointly employ SAM2-based segmentation and VLM-based automatic reporting for pneumothorax detection are quite limited. For this reason, in this study, a holistic system is proposed that combines U-Net for initial segmentation, SAM2 for boundary refinement, and a VLM for automatic clinical interpretation generation.

The main contributions of this study, which directly address the gaps identified above, are summarized below:

1. A two-stage segmentation pipeline is proposed that combines U-Net-based initial segmentation with SAM2-based boundary refinement for pneumothorax.
2. A VLM-based automatic reporting module that converts segmentation outputs into a structured radiological report is designed; its rule-based numerical layer is validated as reproducible, while its free-text layer is explicitly evaluated in Section 3.3 and shown to require radiologist verification before any clinical use (see Section 4).
3. A combined confidence coefficient that integrates the U-Net and SAM2 confidence values is defined and included in the reports.
4. Segmentation performance is evaluated with quantitative metrics, and the automatic reporting outputs are examined through a qualitative case review by expert radiologists on six sample cases, which is reported as a preliminary case-level check and not as a substitute for the systematic BLEU/ROUGE/Likert evaluation specified in Section 3.2.
5. Segmentation performance is reported separately for both all patches (screening performance) and only pneumothorax-containing patches (lesion-level boundary quality), providing an evaluation framework for interpreting overall Dice values in datasets with sparse lesions.

## 2. Materials and Methods

In this study, a holistic approach is proposed for pneumothorax detection, consisting of U-Net-based initial segmentation, SAM2-based segmentation refinement, and VLM-based automatic radiological reporting. The system essentially comprises four stages: image preprocessing, initial segmentation, segmentation refinement, and automatic clinical reporting. The overall workflow is shown in Figure 2.

All figures were prepared with English labels in accordance with international publication standards. In the flow diagram presented in Figure 2, raw CT slices are divided into 224×224 patches from the original resolution without downscaling and are normalized using ImageNet statistics. A bounding box is derived from the region covered by the initial binary mask obtained from the U-Net model and is fed to the prompt encoder of the SAM2 architecture as a box prompt; mask prompts or point prompts were not used in this study. The image encoder, prompt encoder, and mask decoder components within SAM2 operate with frozen weights and combine the original image with the box prompt to generate candidate masks. The U-Net and SAM2 outputs are combined using the agreement rule shown in the diagram to obtain the refined pneumothorax mask. This output, together with the geometric features of the pleural air space (location, extent, and area ratio), is passed to the reporting module; the module separately reports a rule-based numerical layer and a free-text layer generated by a frozen VLM.

### 2.1. Dataset

The dataset used in this study consists of computed tomography (CT) slices provided by the Department of Radiology at Fırat University. The data collection process was carried out in accordance with ethics committee approval and institutional permissions. All images were independently evaluated by three expert radiologists and verified by consensus. However, the detailed annotation protocol is not further specified in this study: it is not recorded how disagreements were adjudicated or what the inter-reader agreement (e.g., a Dice or kappa statistic between annotators) was for the pneumothorax masks specifically; this limits how the lesion-level Dice reported in Section 3 (0.6215 on test) should be interpreted, since it is not known how it compares to a human-to-human annotation-agreement ceiling for this thin, often subtle structure. Three-class (background/pneumothorax/lung) label maps corresponding to the images were created for segmentation; only the pneumothorax class was taken as the binary segmentation target (see Figure 1). The dataset was divided into training, validation, and test subsets. The distribution is given in Table 2.

The distribution given in Table 2 was constructed on a patient basis: the 852 raw image-mask pairs obtained from a total of 171 patients were divided into training (111 patients), validation (17 patients), and test (43 patients) sets such that no patient appeared in more than one set. Each raw image-mask pair was divided into 224×224 patches from the original resolution, yielding a total of 15,628 patches; 73 patches corresponding to empty/black corners were excluded from the dataset, leaving 15,555 patches for the study. Among the generated patches, the proportion of pneumothorax-positive patches was calculated as 13.5% (2105/15,555). During model training, data augmentation (horizontal flipping, rotation of ±10 degrees, and brightness/contrast variation) was applied on-the-fly only to patches in the training set, while the raw class distribution in the validation and test sets was preserved unchanged in order to remain faithful to clinical reality.

### 2.2. Preprocessing

Several preprocessing steps were applied prior to training in order to improve model performance. In the first stage, the images and their corresponding masks were divided into 224×224-pixel patches without downscaling, preserving their original resolution. Downscaling the entire slice to 224×224 in a single step was avoided, as this would cause small structures such as the thin pleural line to disappear. This resolution was chosen because it is compatible with the input size of the MobileNetV2 encoder, reduces computational cost, and allows comparability with previous studies. The effect of resolution on the delineation of the thin pleural line is discussed as a limitation in Section 4.

Pixel-intensity normalization was applied to the images. Pixel values were normalized channel-wise according to ImageNet pretraining statistics using Equation (1).

(1) $$x_{\mathrm{norm}}=\frac{\left(x-\mu \right)}{\sigma }$$

Here, x denotes a pixel value in the image, and μ and σ denote the channel-wise mean and standard deviation values corresponding to the ImageNet-pretrained weights (μ = [0.485, 0.456, 0.406], σ = [0.229, 0.224, 0.225]). In addition, data augmentation was applied to improve the generalization ability of the model. These operations include random rotation of ±10 degrees, horizontal flipping, and brightness/contrast variation. Data augmentation was applied only to the training set.

### 2.3. Initial Segmentation with U-Net

The U-Net architecture was employed for the initial segmentation [13]. U-Net consists of an encoder and a decoder section. The encoder extracts multi-level features from the image, while the decoder reconstructs these features to produce segmentation masks. Skip connections preserve low-level spatial information, preventing the loss of small anatomical structures [14]. In this study, MobileNetV2 [39], pretrained on ImageNet, was used as the encoder. The training parameters are given in Table 3.

MobileNetV2 [39] was preferred over heavier backbones such as ResNet [15], EfficientNet [18], or ConvNeXt [40], and over a plain (non-pretrained) U-Net encoder, primarily for reasons of computational efficiency at patch scale. The 852 raw slices in this study yield 15,555 patches of 224×224 pixels (about 18 per slice; Table 2), so the encoder is invoked once for each of the 9939 training patches per epoch and, at inference, once per patch; MobileNetV2′s inverted residual/depthwise-separable design keeps the encoder to 2,223,872 trainable parameters (Appendix A, Table A1), which is substantially lighter than standard ResNet-50 or EfficientNet-B0 encoders, while its ImageNet-pretrained features are expected to transfer reasonably to small-structure biomedical segmentation. This choice keeps training feasible on a single NVIDIA Tesla T4 GPU (NVIDIA Corporation, Santa Clara, CA, USA; Section 2.7) and keeps per-patch inference latency low enough for the patch-wise screening use case described in Section 4. A systematic head-to-head ablation of MobileNetV2 against ResNet, EfficientNet, ConvNeXt, and non-pretrained U-Net encoders under identical training settings was not performed in this study and is identified as a specific limitation (Section 4) and a concrete direction for future work.

The full layer configuration of the model follows the standard U-Net encoder–decoder arrangement. A detailed layer-by-layer breakdown is presented in Appendix A so as not to disrupt the flow of the main text.

### 2.4. Segmentation Refinement with SAM2

After initial segmentation, a bounding box was derived from the binary mask produced by U-Net and passed as a prompt to the Segment Anything Model 2 system [20]. SAM2 consists of an Image Encoder, a Prompt Encoder, and a Mask Decoder component. If the U-Net mask was not empty, a box was constructed by adding a 10-pixel margin around the pixels covered by the mask. If the U-Net mask was entirely empty, a large box covering the central region of the image (the 5–95% range of the image dimensions) was used.

In this study, the SAM2 module was used with frozen, pretrained weights, in a zero-shot manner, and with only a box prompt. The mask decoder and prompt encoder layers were not additionally fine-tuned on the dataset. The SAM2 mask decoder produces multiple candidate masks (multimask output) for a given prompt, from which the one with the highest quality score is selected. The U-Net and SAM2 outputs do not directly replace one another but are combined using an agreement rule: if the intersection-over-union (IoU) between the two masks falls below a threshold of 0.3, the SAM2 output is considered unreliable and only the U-Net mask is used. If it is above the threshold, the union of the two masks is taken as the final mask. In addition, it was observed that when U-Net did not detect pneumothorax (i.e., produced an empty mask), giving SAM2 a box prompt covering a large part of the image could lead to false-positive segmentation of irrelevant structures such as the chest wall or lung border. For this reason, when U-Net makes a negative decision, an empty mask is returned directly, regardless of the SAM2 output.

### 2.5. Automatic Reporting with VLM

In the final stage, the segmentation outputs obtained with SAM2 are passed to the Vision-Language Model-based reporting module [24]. Two types of information are combined in this module. The first group comprises quantitative fields calculated in a rule-based manner from the mask geometry. The location of the pneumothorax (right or left), its extent, and the estimated area ratio are obtained directly through connected-component analysis and area calculation on the binary mask. Side assignment is based on whether the mask is concentrated in the left or right half of the image frame; no separate radiological/anatomical transformation based on the DICOM orientation metadata of the images is applied. Because laterality errors are a recognized patient-safety concern in radiology reporting, and because Figure 10 shows the system’s own free-text layer disagreeing with its rule-based layer on side, this is a correctness issue that should be resolved before any clinical use, not merely a deferrable limitation (see Section 4). In this study, all side expressions are given based on the left/right half of the image frame. When the combined confidence coefficient is 0.75 or above, the finding is classified as “consistent with pneumothorax”; between 0.55 and 0.75, as “suspicious”; and below this value, as low-confidence. If the refined mask’s area ratio falls below 0.003 (normalized to patch area), the case is classified as “no significant segmentation detected” regardless of the confidence score (consistent with the area ratios of 0.0014 and 0.00065 reported in Figures 7 and 8, which fall below this threshold). The second group consists of free-text descriptions generated by feeding these quantitative fields together with the segmentation image into a general-purpose, open-source Vision-Language Model (Qwen/Qwen2-VL-2B-Instruct [35]). The VLM component used was not additionally fine-tuned on medical images and text; it was used with frozen, pretrained weights and was guided only through a structured prompt template. The free text was obtained via sampling-based generation (temperature 0.6, top-p 0.9, repetition penalty 1.3, maximum 400 new tokens), and it can therefore vary from run to run for the same input. Owing to this separation, the numerical fields are computed reproducibly, while the textual interpretation is generated freely by the language model. In the prompt content, the location, extent, and area ratio of the pneumothorax, along with the U-Net/SAM2/combined confidence scores, are presented to the model as input, and the model is asked to generate a clinical interpretation containing an assessment, treatment-related decision support, and a conclusion section. A fixed disclaimer note is appended to the end of every generated report, stating that the output is intended for decision-support purposes and that the free-text section may contain statements that do not correspond to the image or the numerical findings (risk of hallucination). The reports also present a combined confidence coefficient that integrates the U-Net and SAM2 confidence values. This coefficient is defined by Equation (2). The reporting process is summarized in Figure 3.

*C* = *w*_U_ · *S*_U-Net_ + *w*_S_ · *S*_SAM2_ + *w*_A_ · *A*_ratio_ (2)

Here, *S*_U-Net_ denotes the maximum value in the probability map produced by the U-Net model for foreground pixels, and *S*_SAM2_ denotes the quality score returned by the SAM2 mask decoder for the selected mask (the former is a sigmoid probability, the latter a quality score). The weights *w*_U_, *w*_S_, and the area-term weight were chosen such that they sum to 1, and in this study *w*_U_ was set to 35%, *w*_S_ to 45%, and *w*_A_ (the area-term weight) to 20%. *A*_ratio_ is the area ratio of the refined mask normalized to 3% (with an upper bound of 1.0), which prevents very small/noisy segmentations from being reported with a high confidence score. This confidence value is a quantity distinct from the Dice coefficient, which measures overlap with the ground-truth mask. Therefore, observing a high confidence value together with a moderate Dice value does not constitute a contradiction. In addition, the combined confidence coefficient is also computed numerically when the segmentation area remains below the threshold and a “no significant segmentation detected” decision is made; however, in this case it carries no evidentiary value regarding the presence of a finding. For negative decisions, the coefficient should be reported for informational purposes only.

The selection of the weights as *w*_U_ = 0.35, *w*_S_ = 0.45, and *w*_A_ = 0.20 is based on a heuristic rationale grounded in the characteristic predictive behavior of the two models, rather than on a systematic grid search or ablation study. The U-Net model exhibits a more stable behavior in pixel-wise probability distributions (sigmoid probabilities) and shows consistency in capturing the general outlines of anatomical structures; its contribution to the confidence score was therefore set to 35%. The SAM2 architecture, owing to its multi-scale feature extraction, was weighted slightly higher at 45%, based on the expectation, not confirmed by the results reported here, that it can delineate fine boundaries and the pneumothorax-specific pleural line more sharply. In addition, a regularizing term (*A*_ratio_) based on the area ratio of the refined mask was added, with a weight of 20%, to prevent very small or anatomically inconsistent regions from being reported with a high confidence score. The correlation of these weights with accuracy or clinical appropriateness has not been separately validated within the scope of this study, and a systematic sensitivity analysis is proposed as future work in Section 4.

A concrete sensitivity-analysis protocol is proposed as the next step: (i) a grid search over *w*_U_ and *w*_S_ in increments of 0.05, subject to *w*_U_ + *w*_S_ + *w*_A_ = 1 and *w*_A_ held in the 0.10–0.30 range, re-scoring the six qualitative cases in Section 3.3 together with the full test set; (ii) a calibration curve comparing the combined confidence coefficient against radiologist-confirmed outcomes, binning predictions and checking whether nominal confidence tracks observed agreement; and (iii) an ablation in which each term (*S*_U-Net_, *S*_SAM2_, *A*_ratio_) is individually removed (weight set to zero and the remainder renormalized) to quantify its marginal contribution to case-level ranking. Because *S*_U-Net_ was observed to saturate at its upper bound (1.0) in the worked examples (Figures 7–12), this protocol would also clarify whether *w*_U_ should be reduced in favor of *w*_S_ or *w*_A_ once a larger radiologist-labeled validation set is available; it is not applied in the present study, which relies on the heuristic weighting described above.

### 2.6. Evaluation Metrics

Segmentation performance was evaluated using the Accuracy, Dice coefficient, IoU, and Loss metrics. Denoting the predicted mask set as A and the ground-truth mask set as B, the Dice coefficient is calculated with Equation (3), IoU with Equation (4), and Accuracy with Equation (5).

Dice = 2|A ∩ B| / (|A| + |B|)(3)

IoU = |A ∩ B| / |A ∪ B|(4)

Accuracy = (TP + TN) / (TP + TN + FP + FN)(5)

Here, TP, TN, FP, and FN denote the numbers of true-positive, true-negative, false-positive, and false-negative pixels, respectively. Sensitivity is calculated with Equation (6) and Specificity with Equation (7).

(6) $$\mathrm{Sensitivity}=\mathrm{TP}\frac{}{}\left(\mathrm{TP}+\mathrm{FN}\right)$$

(7) $$\mathrm{Specificity}=\mathrm{TN}\frac{}{}\left(\mathrm{TN}+\mathrm{FP}\right)$$

For a more detailed assessment of boundary quality, the 95th-percentile Hausdorff distance (HD95) was additionally reported. HD95 was calculated in pixel units as the 95th percentile of the mutual nearest-point distances between the predicted and ground-truth mask boundaries, and by definition it is only defined for samples in which neither mask is empty. For patch-level screening metrics, a patch was considered positive if its ground-truth mask contained at least one pneumothorax pixel, and was considered predicted positive if its predicted mask contained at least one positive pixel.

It is planned to also evaluate the performance of the automatic reporting module in terms of text quality. The overlap between the generated reports and reference radiologist interpretations is intended to be measured using BLEU and ROUGE metrics, clinical appropriateness through radiologist Likert ratings, and inter-observer agreement through Cohen’s kappa coefficient (see Section 3.2). In addition to this planned systematic evaluation, a qualitative assessment was also carried out by a panel of expert radiologists on sample cases (see Section 3.3).

### 2.7. Implementation Details

Training was carried out on an NVIDIA Tesla T4 GPU. The U-Net model was trained for 50 epochs with a batch size of 16 and an initial learning rate of 0.0001 using the Adam optimization algorithm [22]. The learning rate was scheduled to be halved when the validation loss plateaued. The equally weighted average of the Dice loss and the Binary Cross-Entropy loss (0.5 × Dice + 0.5 × BCE) was used as the loss function. The implementation was built on the segmentation_models_pytorch library (version 0.5.0); the sam2.1_hiera_small checkpoint was used for SAM2, and the transformers library (version 5.13.1) was used for the VLM. For reproducibility, the Python, NumPy, and PyTorch random seeds were fixed at 42. Training was carried out with Python 3.12 and PyTorch 2.11, and the total training time for U-Net was measured at approximately 4.5 h. The SAM2 module was not additionally trained; it was used solely for inference with frozen, pretrained weights.

## 3. Results

The segmentation performance of the proposed system was evaluated using the Accuracy, Dice coefficient, IoU, and Loss metrics. The results of the initial U-Net segmentation and the SAM2-based refinement are compared in Table 4. The table includes both validation and test set results; HD95 was calculated only over samples in which both the ground-truth mask and the prediction were non-empty, and empty-empty pairs and entirely missed samples were not included in this metric.

Examination of Table 4 shows that SAM2-based boundary refinement, used with frozen weights without fine-tuning, does not provide a clear advantage over U-Net initial segmentation, with the two methods showing comparable performance. While the U-Net validation Dice value was 0.9309, this value remained almost unchanged at 0.9325 after SAM2. On the independent test set, U-Net alone achieved the highest performance with a Dice of 0.9368 and an IoU of 0.9279, while SAM2 refinement produced a close result of 0.9244 Dice. On the test set, U-Net also yielded the lowest boundary error with an HD95 of 27.53 pixels, with the SAM2 output remaining close at 29.36 pixels. The increase in the SAM2 HD95 value (51.84 pixels) relative to the U-Net value (34.81 pixels) on the validation set indicates that boundary refinement did not provide a systematic gain on this dataset. The closeness of the training and validation results indicates that the U-Net model does not exhibit overfitting behavior. This finding suggests that, once a strong initial segmentation is obtained, the contribution of boundary refinement with frozen SAM2 remains limited in regional overlap metrics such as Dice/IoU, and that the SAM2 contribution may be more oriented toward regularizing mask boundaries and cleaning up noisy regions (see Section 4).

To contextualize these headline Dice values, it is useful to compare them against a degenerate baseline that always predicts an empty mask. Because Dice equals 1.0 by definition when both the ground truth and the prediction are empty, and negative patches make up approximately 86.5% of the data, such a trivial predictor would already score 3483/3991 = 0.8727 Dice on the test set and 1411/1625 = 0.8683 on validation, without segmenting a single pixel. The reported test Dice of 0.9368 is therefore only 6.4 percentage points above this degenerate baseline, and aggregate Dice over all patches is consequently driven mainly by the negative-patch true-negative rate rather than by boundary-overlap quality. This is precisely why Table 5, which restricts the calculation to ground-truth-positive patches (Dice 0.6215 on test), and not the all-patch Dice in Table 4, is the quantity that should be treated as the primary measure of segmentation (boundary-delineation) quality; the all-patch Dice is better read as a screening-level agreement statistic.

Two further mechanisms plausibly explain why the fused U-Net + SAM2 output (reported in the “U-Net + SAM2” columns of Table 4; see Section 2.4 for the exact fusion rule) does not exceed U-Net alone. First, the fusion rule takes the pixel-wise union of the two masks whenever their IoU exceeds 0.3, and a union is, by construction, monotonically non-decreasing in area; the fused mask can therefore only stay the same size or grow relative to each individual mask, which biases it toward over-segmentation and a corresponding loss of precision. The slightly lower test-set Dice after fusion is consistent with this precision loss, although the present study does not separately decompose precision and recall to confirm it directly, which is noted as a limitation below. Second, the empty-mask override (Section 2.4) returns an empty mask outright whenever U-Net’s own prediction is empty, regardless of what SAM2 would have proposed; this means the SAM2 stage can only ever modify a patch that U-Net has already flagged as positive, and can structurally never recover a patch that U-Net missed. The fusion stage is therefore incapable, by design, of improving sensitivity, and any claimed benefit of SAM2 refinement is necessarily confined to reshaping the boundary of already-detected regions rather than to detecting additional true positives.

The Loss, Dice, IoU, and Accuracy curves obtained during training are presented together in a single combined panel in Figure 4.

Examination of the curves reveals rapid learning behavior in the initial epochs, with the curves becoming stable after approximately epoch 15–20. The fact that the validation Dice coefficient reached 93.09%, the validation IoU 92.09%, and the validation Accuracy 99.71% shows that the U-Net model can reliably distinguish between patches with and without pneumothorax; the within-lesion boundary accuracy is separately given in Table 5 and is more moderate. The absence of a clear divergence between the training and validation curves indicates strong generalization behavior.

A large part of the overall Dice values in Table 4 results from the model correctly producing an empty prediction on patches that do not contain pneumothorax (GT-negative); these patches constitute approximately 86.5% of the total patches. Table 5 shows the segmentation quality calculated only on patches containing pneumothorax (GT-positive). Accordingly, the Dice value in true lesion regions is 0.6295 on validation and 0.6215 on the test set; this shows that the overall Dice value of around 0.93 in Table 4 largely reflects screening performance, whereas the regional overlap quality is more modest. In contrast, patch-level screening performance is high: 82.2% (validation) and 79.9% (test) of positive patches are correctly detected (sensitivity), and more than 97% of negative patches are correctly classified as empty (specificity). This finding indicates that the system performs strongly as a clinical screening tool but shows a more limited performance as a pixel-level boundary-delineation tool; a comparison of the two measurement levels is presented visually in Figure 5.

### 3.1. Comparison with Literature

A comparison of the proposed method with pneumothorax segmentation studies in the literature is given in Table 6.

In the proposed method, the U-Net component alone yielded a numerically higher Dice value (0.9368), computed over all patches, than the values reported in the literature. Although SAM2-based boundary refinement did not provide an additional gain in Dice/IoU on this dataset (0.9244/0.9129), no boundary-regularity metric was computed to support a claim that it produces more regular mask boundaries around the pleural line, and the validation HD95 value in fact worsens after fusion (Table 4); any boundary-regularization benefit of SAM2 therefore remains an unconfirmed hypothesis rather than an established finding of this study. It should be emphasized that the comparison in Table 6 is made across different imaging modalities (chest radiography and CT) and different datasets, and the claim of numerical superiority should therefore be regarded as indicative only. In addition, the metric definition also differs: the compared studies report the Dice value over lesion-containing samples, whereas the 0.9368 value in this study also includes patches without pneumothorax. The quantity closest to a direct comparison is the lesion-level Dice value given in Table 5 (0.6215); this value is given as a separate row in Table 6.

### 3.2. Report Evaluation

It was planned to measure the quality of the automatically generated reports using automatic text-overlap metrics (BLEU, ROUGE) and expert evaluation (Likert ratings, and Cohen’s kappa for inter-observer agreement). However, calculating these metrics requires a dataset containing reference reports written by radiologists. Since radiologist reference reports and ratings had not yet been collected within the scope of this study, numerical scores for BLEU, ROUGE, Likert, and Cohen’s kappa are not reported. These measurements are addressed as a limitation in Section 4 and are planned as future work. Instead, the system’s outputs were qualitatively evaluated on sample cases by a panel of the three expert radiologists involved in the study; the findings of this case-level assessment are presented in Section 3.3.

The planned quantitative evaluation is specified as follows so that it can be executed as a direct continuation of the present study. A held-out set of at least 100 CT slices, stratified across the six clinical patterns illustrated in Section 3.3 (unremarkable, small/insignificant air trace, and confirmed pneumothorax of varying size), will be independently reported in free text by the three radiologists involved in this study to serve as reference reports. System-generated reports will then be scored against these references using BLEU-4 and ROUGE-1/ROUGE-L for lexical overlap, while clinical appropriateness will be rated independently by each radiologist on a 5-point Likert scale (1 = clinically misleading/unsafe, 3 = acceptable with reservations, 5 = fully consistent with the reference report), with inter-rater reliability summarized by Cohen’s kappa (and Fleiss’ kappa across all three raters). This protocol directly targets the hallucination cases documented in Section 3.3 and Section 4, since a report such as the laterality mismatch in Figure 6 would be expected to receive a low Likert score despite plausible-sounding language.

### 3.3. Qualitative Results

Figure 6 shows a typeset example of the automatic report generated by the system for the case shown in Figure 10: the rule-based numerical findings, the free text generated by the VLM, and the fixed disclaimer note are presented as separate sections. The fact that the side (right) stated in the free-text section contradicts the rule-based location (left) is a concrete example of the hallucination tendency discussed in Section 4.

All side (right/left) expressions in this section were generated based on the image frame, as defined in Section 2.5; definitive side assignment according to patient anatomy requires confirmation of the relevant slices against the DICOM orientation metadata (see Section 4). Below, actual outputs obtained by running the proposed system through its Gradio-based interface on six different CT slices are presented. In Figure 7, Figure 8, Figure 9, Figure 10, Figure 11 and Figure 12, the input slice, the segmentation overlay, and the report output generated by the system are presented together in a single figure for each example; the radiologist assessments are given as text below the figures. The rule-based findings for each example (Conclusion, Location, Extent, area ratio, and confidence scores) were separately evaluated and verified by comparing them with the corresponding CT slice by the expert radiologist involved in the study. In Figure 7, Figure 8, Figure 9 and Figure 11, the radiologist likewise did not confirm a clinically significant air collection in the pleural space; in Figure 10 and Figure 12, a clear pneumothorax finding, in terms of size and morphology, was confirmed in the left hemithorax. The free-text interpretations generated by the VLM are briefly summarized below each example; these summaries reflect concrete examples of the hallucination tendency discussed in detail in Section 3.2 and Section 4, and do not substitute for a clinically verified radiologist assessment.

Assessment of the VLM free text for Figure 7. The VLM referred to an ill-defined textural change in the left upper region but used a fabricated term with no medical equivalent to describe the finding, and, avoiding a definitive assessment, listed generic, non-specific recommendations.

Radiologist assessment for Figure 7. In this slice, a millimetric, ill-defined air density was observed adjacent to the trachea, at the right paratracheal level. The radiologist’s assessment indicated that this appearance was consistent with a partial-volume effect of normal anatomical structures at this level (the trachea and adjacent vascular/tissue borders) rather than a true air collection in the pleural space; a clinically significant pneumothorax finding was not confirmed. This assessment, consistent with the system’s very low area ratio (0.0014), supports the correctness of the “no significant segmentation detected” decision given the size of the finding, even though the combined confidence coefficient (0.7127) lies in the “suspicious” band (0.55–0.75) defined in Section 2.5.

Assessment of the VLM free text for Figure 8. The VLM interpreted the marked area in the left upper region as a “tumor”, unrelated to pneumothorax, and generated off-topic oncological statements such as malignancy risk and treatment options; this interpretation does not correspond to the numerical findings.

Radiologist assessment for Figure 8. In this slice, an ill-defined air trace, even smaller than the previous one (area ratio 0.00065), was marked at the right upper mediastinal border. The radiologist’s assessment indicated that, given its size and location, this finding was consistent with measurement/segmentation noise and did not reflect a true pleural air collection. This example represents a case in which the system’s classification of very small, clinically insignificant findings as “not detected” was appropriate.

Assessment of the VLM free text for Figure 9. Rather than associating the marked region with pneumothorax, the VLM referred to a “spinal sinus” and an associated fictitious syndrome; the generated text is anatomically inconsistent and does not correctly describe the finding in the image.

Radiologist assessment for Figure 9. In this slice, a marking was made on a small, irregularly bordered structure at the level of the neck base/thoracic inlet. The radiologist’s assessment indicated that this area corresponded to a vascular/lymphatic tissue border and did not show an air-tissue distinction consistent with pneumothorax. This example shows that, at slices around the level of the upper thoracic inlet, the model can sometimes mark anatomical structures with low confidence, but that the system’s final “Conclusion” decision was consistent with the radiologist’s assessment at this level.

Assessment of the VLM free text for Figure 10. The VLM described the marked area as a large air space but stated the side (right) in a manner that contradicted the rule-based finding (left); it used non-specific, generic statements in the treatment and conclusion sections.

Radiologist assessment for Figure 10. The radiologist’s assessment confirmed that, in this slice, there was an avascular, air-density area at the apex of the left lung, clearly demarcated from the adjacent lung parenchyma; the appearance was small in size but morphologically consistent with a typical apical pneumothorax. It was noted that the segmentation boundaries correctly followed the pleural line. The high individual confidence scores of U-Net and SAM2 (1.0 and 0.8494, respectively) and the resulting combined confidence coefficient of 0.79 are consistent with the finding confirmed by the radiologist.

Assessment of the VLM free text for Figure 11. As in the previous examples, the VLM interpreted the finding as a tumor formation and referred to staging and treatment approaches; this assessment is unrelated to the pneumothorax finding and is not supported by the numerical findings.

Radiologist assessment for Figure 11. In this slice, a small, triangular air trace was marked between the trachea and the apex of the right lung. As in the previous examples, the radiologist’s assessment confirmed that, given its size and location, this finding did not constitute a clinically significant pneumothorax and was consistent with normal anatomical boundaries.

Assessment of the VLM free text for Figure 12. Unlike Figure 7, Figure 8, Figure 9, Figure 10 and Figure 11, the VLM output for this case was largely incoherent rather than a well-formed but clinically false statement: it referred to an undefined numeric value (“approximately −5”) with no clear clinical referent, and its treatment-support and conclusion sections were generic and did not form a coherent clinical argument, at one point introducing an unrelated term with no connection to the image or the numerical findings. The free text also stated the side as right, contradicting the rule-based location (left)—the same laterality-mismatch pattern seen in Figure 10.

Radiologist assessment for Figure 12. The radiologist’s assessment confirmed that, in this slice, there was a large, crescent-shaped, air-density area in the upper-outer part of the left hemithorax, clearly demarcated from the lung parenchyma. It was noted that the segmentation boundaries correctly followed the lung margin. The high U-Net and SAM2 confidence scores (1.0 and 0.8182) and the combined confidence coefficient of 0.7866 are consistent with this radiologist assessment.

As seen in the six examples above, the rule-based numerical findings (Conclusion, Location, Extent, area ratio, confidence scores) were computed consistently and reproducibly in every case and were verified by the expert radiologist in the study by comparison with the corresponding CT slices. In four examples (Figure 7, Figure 8, Figure 9 and Figure 11), small findings consistent with normal anatomical boundaries were considered clinically insignificant; in two examples (Figure 10 and Figure 12), clear pneumothorax findings, in terms of size and morphology, were confirmed in the left hemithorax. Although this case-level qualitative verification provides supporting evidence for the clinical validity of the system’s rule-based layer, it does not substitute for the systematic BLEU/ROUGE/Likert evaluation described in Section 3.2. The free-text component generated by the VLM continues to carry a risk of hallucination, as discussed in detail in Section 4, and it is emphasized that this component should not be used in clinical decision-making without radiologist verification.

### 3.4. Ablation Analysis of the SAM2 Refinement Module

This section treats the U-Net-only configuration and the full U-Net + SAM2 pipeline reported in Table 4 and Table 5 as two arms of an ablation in which the only variable is whether the SAM2 box-prompted refinement and agreement rule (Section 2.4) are applied after U-Net’s prediction. The comparison is indicative rather than paired: the U-Net columns use the complete validation and test sets, whereas the U-Net + SAM2 columns use a random subset of about 300 patches (Table 4 caption). On overall (screening-level) metrics, removing SAM2 changes test Dice by only +0.0124 (0.9368 vs. 0.9244) and test IoU by +0.0150 (0.9279 vs. 0.9129), i.e., SAM2 does not yield a quantitatively meaningful gain in regional overlap on this dataset. On the boundary-specific HD95 metric, the ablation is more informative: on the validation set, adding SAM2 increases HD95 from 34.81 to 51.84 pixels (worse), whereas on the test set it increases HD95 from 27.53 to 29.36 pixels only marginally; taken together, the boundary evidence does not support a systematic gain from SAM2 refinement on this dataset either, which is consistent with, and quantifies, the qualitative impression in Section 3.1.

This ablation isolates one design axis only: SAM2 present vs. absent, with SAM2 used in its default zero-shot, frozen-weight, box-only prompt configuration (Section 2.4). It does not vary the IoU agreement threshold (fixed at 0.3), the 10-pixel box margin, or the prompt type (box vs. point vs. mask), each of which is a plausible source of the limited measured gain and each of which would require its own controlled runs; these parameter-level ablations were not performed in the present study and are listed as a concrete methodological limitation in Section 4. The available evidence supports only the narrower conclusion that, under a strong U-Net initial segmentation, frozen zero-shot SAM2 refinement did not yield a consistent Dice/IoU gain; whether it provides any boundary-regularization benefit remains untested and should be re-examined once SAM2 is either fine-tuned on this dataset or driven with denser prompts.

## 4. Discussion

In this study, a holistic approach was proposed for pneumothorax detection, combining U-Net-based initial segmentation, SAM2-based segmentation refinement, and VLM-based automatic reporting. The experimental results show that the proposed method can distinguish patches containing pneumothorax with high sensitivity and specificity, while delineating lesion boundaries with moderate accuracy.

The fact that the Dice coefficient, IoU, and Accuracy of the U-Net-based initial segmentation on the validation set were 93.09%, 92.09%, and 99.71%, respectively, indicates a high degree of overlap between the predicted segmentation regions and the ground-truth masks. On the independent test set, these values were found to be 93.68%, 92.79%, and 99.79%, respectively. However, since these values were computed over all patches, they largely reflect screening performance; on patches containing pneumothorax only, the Dice value remains at 0.6295 on validation and 0.6215 on the test set (Table 5). This distinction reveals that the system’s lesion-capture (screening) ability and its pixel-level boundary accuracy are quantities of a different order. The small difference between the training and validation/test results indicates that there is no overfitting problem and that the model generalizes well to images from different patients.

Several factors plausibly explain why lesion-level Dice remains moderate even though patch-level screening is reliable. First, the pathology itself is thin: a pneumothorax appears as a sliver of air along the pleural line that can be only a few pixels wide at 224×224 resolution, so a small absolute boundary error translates into a large relative overlap penalty even when the finding is correctly localized. Second, positive patches are rare (13.5% of all patches, Table 2), so the training signal for precise boundary delineation is comparatively sparse relative to the signal for the easier positive/negative screening decision. Third, partial-volume effects and small vascular or anatomical structures near the pleural line (illustrated by the borderline cases in Section 3.3) make boundaries genuinely ambiguous even for an expert radiologist, which can inflate boundary disagreement without reflecting a true model failure. Fourth, because patches are segmented independently (Section 2.2), a pneumothorax rim that straddles a patch boundary is never seen in full spatial context by the encoder, which can truncate or fragment the predicted boundary relative to the continuous ground-truth rim. These mechanisms are complementary rather than mutually exclusive, and distinguishing their relative contribution would require a dedicated error-analysis study stratifying failures by lesion thickness, patch-edge proximity, and reader agreement, which is proposed as future work.

Although high accuracy has been reported in the literature for U-Net-based pneumothorax segmentation studies, boundary losses and false-positive results can be observed in small pneumothorax regions [14,29]. EfficientNet-based U-Net approaches have been shown to increase feature-extraction capacity [18]. Sae-Lim et al. strengthened quantitative assessment with a two-stage approach [30]. In this study, adding boundary refinement with frozen SAM2 after obtaining a strong initial U-Net segmentation did not provide a marked additional gain in regional overlap metrics such as Dice and IoU, and slightly increased the HD95 value on the validation set. This finding suggests that the SAM2 contribution may remain limited to qualitative aspects such as boundary regularity and noise cleaning when U-Net is already strong, and that SAM2-based refinement may provide a more pronounced benefit particularly in cases where U-Net is weaker and boundary uncertainty is higher [20,21].

One of the important contributions of the proposed method is that it does not perform segmentation alone but can also generate automatic radiological interpretations using a VLM. A substantial portion of the studies in the literature perform only classification or segmentation [29,33]. In addition to identifying the pathological region, the system developed in this study can also generate clinical explanations. Examination of the automatically generated interpretations shows that the explanations occasionally have a structure resembling radiological terminology, but, as demonstrated in Section 3.3, they can also contain statements that do not correspond to the image or have no medical equivalent. For this reason, the potential of the free-text component to shorten expert evaluation time in institutions with a heavy patient load [24,34] should be considered only under the condition of medical-domain-specific fine-tuning and radiologist oversight. Adding methods such as Grad-CAM [8] and SHAP [9] to strengthen the explainability of model decisions may be considered in future work.

Several concrete mitigation strategies follow directly from the hallucination examples documented in Section 3.3. (i) Rule-based cross-verification: the numerical fields (location, extent, area ratio) are already computed deterministically (Section 2.5) and could be used to automatically flag or suppress free-text sentences that contradict them, as in the laterality mismatch in Figure 6, rather than only appending a generic disclaimer. (ii) Constrained or template-guided decoding that restricts the VLM to filling predefined clinical slots (location, size category, recommendation) instead of unconstrained free generation, which would reduce the space in which fabricated terms (e.g., the non-medical terms noted in Figure 7 and Figure 9) can appear. (iii) Medical-domain fine-tuning of the VLM component, e.g., in the style of LLaVA-Med [36] or using biomedical vision-language pretraining [34], rather than relying on a general-purpose model used zero-shot. (iv) Lower-temperature or multi-sample majority-vote decoding to improve reproducibility, since the current sampling-based generation (temperature 0.6, Section 2.5) is explicitly non-deterministic. (v) A mandatory human-in-the-loop verification gate before any free-text output reaches a clinical record, which is already recommended in Section 3.3 and Section 4 and is treated here as a hard requirement rather than a soft caveat.

In terms of practical implications, the rule-based numerical layer of the proposed system (location, extent, area ratio, and the combined confidence coefficient), which is reproducible and does not depend on VLM sampling, is the component closest to near-term clinical utility: given its lightweight MobileNetV2-based encoder (Section 2.3) and modest hardware requirement (a single Tesla T4 GPU, Section 2.7), it could plausibly be deployed as a triage-support tool that flags CT series containing a high-confidence pneumothorax finding for prioritized radiologist review in emergency and intensive-care settings, where delayed diagnosis carries direct clinical risk (Section 1). The high patch-level screening sensitivity and specificity (Table 5) make such prioritization plausible even though pixel-level boundary delineation remains moderate; a prioritization use case depends on detecting the presence of pneumothorax, not on delineating its exact boundary. By contrast, the free-text VLM reporting layer, given the hallucination risk documented in Section 3.3, is not yet suitable for unsupervised use and should be positioned as a drafting aid that a radiologist edits and countersigns, not as an autonomous reporting step, until the evaluation protocol in Section 3.2 and the mitigation strategies above have been carried out.

The study has several limitations. First, the single-center design limits generalizability, as discussed in detail below. Second, the 224×224 resolution may be limiting for delineating the thin pleural line. A comparison with higher-resolution inputs is planned for the future. Third, the SAM2 and VLM models require substantial computational power. Optimization with lightweight architectures may be necessary in terms of processing time for real-time clinical use. Fourth, in this study, SAM2 was used with frozen weights and only a box prompt, and it did not provide a consistent gain in the Dice/IoU metrics. The extent to which SAM2 could contribute with fine-tuning or with different prompt strategies (point, mask prompt) requires a separate analysis. In the same vein, the MobileNetV2 encoder was not benchmarked head-to-head against ResNet, EfficientNet, or ConvNeXt under identical training settings (Section 2.3), so its specific contribution relative to these alternatives remains unconfirmed. Fifth, the weights of the combined confidence coefficient (*w*_U_, *w*_S_, *w*_A_) were determined heuristically, and their correlation with accuracy or clinical appropriateness has not yet been validated through a systematic sensitivity analysis. Sixth, the VLM component used (Qwen2-VL-2B-Instruct) is a general-purpose, relatively small-scale model that has not been additionally fine-tuned in the medical domain. As seen in the example outputs presented in Section 3.3, the free-text clinical interpretation generated by this component can occasionally contain inconsistent statements or statements that do not correspond to the image (hallucination); moreover, since the free text is obtained through sampling-based generation, it is not reproducible for the same input. For this reason, only the rule-based numerical findings (location, extent, area ratio, confidence scores) in the system should currently be regarded as reproducible and reliable, and the free-text section should not be used in clinical decision-making without verification by a radiologist. Seventh, no statistical significance test or confidence interval was calculated for the U-Net and SAM2 comparisons reported in the study; therefore, the extent to which the differences in Table 4 are affected by sample noise should be separately assessed. Likewise, precision and recall of the fused mask were not computed separately, so the over-segmentation explanation given in Section 3 remains a hypothesis. This uncertainty is increased by the fact that the SAM2 columns were computed over a subset of approximately *n* ≈ 300 samples containing only about 40 positive patches. Eighth, since side (right/left) assignment is based directly on the image half, the CT export convention used (radiological or anatomical display) needs to be confirmed against the DICOM orientation metadata; it should also be noted that the U-Net term (*S*_U-Net_) in the combined confidence coefficient was found to be saturated at its upper bound (1.0) in the examined examples, which may limit the discriminative power of the coefficient, and that selection of the largest connected component may exclude bilateral pneumothorax cases. Finally, the reference report dataset required to quantitatively validate automatic report quality with BLEU/ROUGE and radiologist Likert scoring has not yet been constructed within the scope of this study. This evaluation is planned as a separate study.

Regarding generalizability, the single-center design (171 patients, Section 2.1) means that scanner protocol, reconstruction kernel, slice thickness, and patient-population characteristics are all confounded with the institution, so the reported Dice/IoU/Accuracy values should be read as an upper bound on performance until confirmed elsewhere; deep-learning segmentation models are well known to lose accuracy under such distribution shift. Consequently, no claim of cross-institution generalization is made in this paper. External validation on at least one additional center’s CT archive, ideally acquired on a different scanner vendor, is identified as the highest-priority next step before any clinical deployment claim, alongside the multicenter surveillance precedent reported for chest-radiograph pneumothorax detection [4].

Regarding patch-level context, dividing each slice into independently processed 224×224 patches (Section 2.2) avoids the loss of thin structures that whole-slice downscaling would cause, but it also means the encoder never observes a pneumothorax rim that straddles a patch boundary in full spatial context, and no explicit cross-patch stitching or boundary-consistency post-processing is applied when patch predictions are reassembled. This is a plausible contributor to the moderate lesion-level Dice discussed above and to isolated boundary fragmentation. Overlapping sliding-window inference with prediction averaging in the overlap region, or a lightweight global-context branch that conditions each patch on coarse whole-slice features, are both concrete directions for reducing this effect and are proposed as future work.

Regarding the U-Net/SAM2 fusion rule, the current design (a fixed IoU-agreement threshold of 0.3 followed by a mask union, Section 2.4) is a simple, interpretable heuristic rather than a learned mechanism, and the ablation in Section 3.4 shows it does not yield a consistent Dice/IoU gain on this dataset. A learned fusion strategy, for example, a lightweight gating or attention module that weighs the two masks per pixel based on each model’s local confidence rather than a single global IoU threshold, is a promising alternative; such a mechanism could be trained once a training signal for the fusion step itself (e.g., from the sensitivity-analysis protocol in Section 2.5) is available. This is noted as a specific, currently unimplemented, future-work item rather than a claim evaluated in the present study.

Regarding clinical size mapping, the area ratio reported by the system (Section 2.5) is computed relative to the pixel area of a 224×224 patch, and no pixel spacing (mm/pixel) is recorded or reported anywhere in this manuscript. Consequently, the area ratio cannot currently be converted to a physical quantity such as millimeters or milliliters, and cannot be mapped to the size-based clinical decision thresholds (e.g., small vs. large pneumothorax, or conservative management vs. aspiration vs. chest-tube drainage) that guide real pneumothorax management. A hemithorax-normalized pneumothorax fraction, using the lung-class label that is already produced by the same three-class annotation protocol (Section 2.1) as the denominator, would be a more clinically interpretable quantity than a raw patch-relative area ratio; the lung class is currently used only for anatomical illustration (Figure 1) and this normalization is proposed as future work rather than implemented in the present study.

Regarding prevalence and patient-level evaluation, the patch-level positive prevalence in the test set is 508/3991 = 12.7% (Table 2), which already reflects a curated research cohort rather than an unselected clinical stream; realistic emergency-triage prevalence for pneumothorax on a given CT slice is likely substantially lower. Because positive predictive value (PPV) depends strongly on prevalence, the same sensitivity/specificity pair implies a much lower PPV at low prevalence than at the prevalence used here (for example, 80% sensitivity and 98% specificity give a PPV of roughly 0.86 at the overall-dataset prevalence of 13.5% (Table 2) but only around 0.45 at 2% prevalence); PPV itself is not reported in this study, and the sensitivity/specificity values in Table 5 should not be read as directly transferable to a lower-prevalence triage setting without a prevalence-matched validation. In addition, all detection metrics in this study are reported at the patch level; no patient-level endpoint (e.g., the number of the 43 test patients with at least one true-positive slice who were correctly flagged, or the number subject to at least one false-positive slice) is currently reported, and the 508 positive test patches likely derive from a considerably smaller number of underlying lesions and patients, so the effective sample size for lesion-level inference is closer to the patient count than to the patch count. A patient-level re-analysis of the existing test set is identified as a concrete, low-cost next step and is left for future work.

In future work, it is planned to conduct a systematic ablation study comparing a fine-tuned version of SAM2 with different prompt strategies, to complete the BLEU/ROUGE and Likert/Cohen’s kappa evaluations by collecting a dataset of radiologist reference reports, to validate the confidence-coefficient weights through a sensitivity analysis, to develop multimodal systems that jointly use different imaging modalities, and to improve reporting performance and consistency by fine-tuning the VLM component on medical text and image data.

## 5. Conclusions

In this study, a holistic automatic radiological reporting system based on U-Net, SAM2, and a VLM was proposed for pneumothorax detection. In the system, initial segmentation was performed with U-Net, after which the segmentation boundaries were refined with SAM2; features extracted from the identified pleural air spaces were then passed to the VLM reporting module.

As a result of the experiments, the validation Dice coefficient, validation IoU, and validation Accuracy were found to be 93.09%, 92.09%, and 99.71%, respectively. On the independent test set, Dice, IoU, and Accuracy were found to be 93.68%, 92.79%, and 99.79%, respectively. When measured only on patches containing pneumothorax, the Dice value was found to be 0.6295 on validation and 0.6215 on the test set; patch-level screening sensitivity was 82.2% (validation) and 79.9% (test), with specificity above 97%. The results show that U-Net-based initial segmentation can reliably distinguish patches containing pneumothorax, while within-lesion boundary accuracy remains open to improvement. Although boundary refinement with frozen SAM2 did not provide an additional gain in Dice/IoU on this dataset (random test subsample of about 300 patches: Dice 92.44%, IoU 91.29%), any boundary-regularization benefit remains untested. The small difference between the training and validation results indicates stable learning behavior. An important aspect of the proposed approach is that findings such as location, extent, and area ratio, computed from the segmentation output, can be automatically generated in a reproducible manner. Although the VLM-based free-text interpretation component has the potential to translate these numerical findings into a clinical narrative, its output consistency is limited in its current form, as it relies on a general-purpose model that has not been fine-tuned, and it requires radiologist verification and quantitative evaluation (BLEU/ROUGE, Likert) prior to clinical use. The developed system, particularly through its rule-based numerical layer, is considered to have the potential to support the preliminary evaluation process in emergency departments and radiology units. In the future, it is aimed to improve system performance and report consistency through different datasets, multimodal imaging, and fine-tuning of the VLM component on medical datasets.

## Figures and Tables

**Figure 1:**
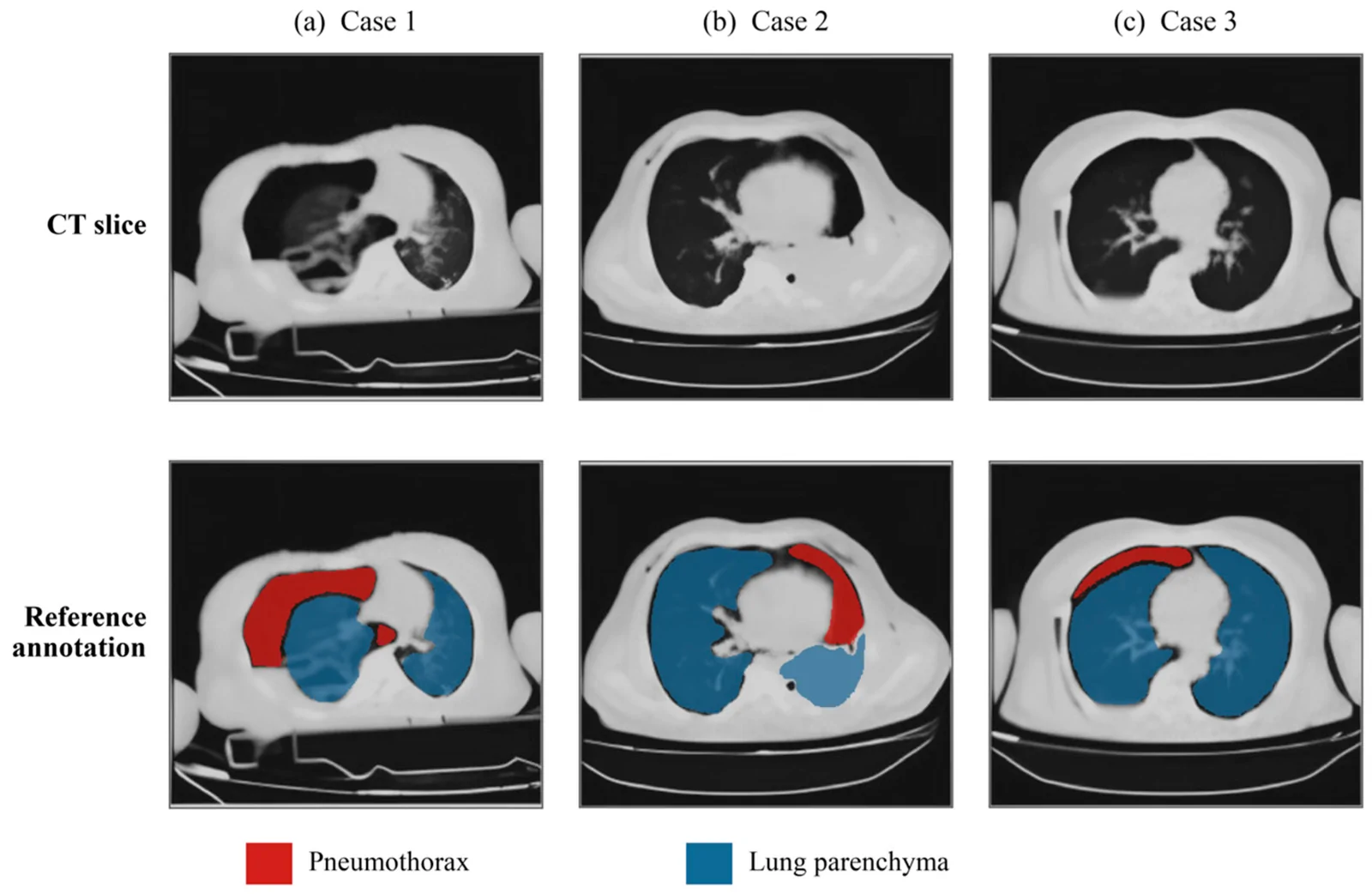
Example computed tomography (CT) slices from the dataset and their corresponding reference annotations. The reference masks are three-class label maps (background/pneumothorax/lung); only the pneumothorax class was used as the binary segmentation target, and the lung class is shown here only for anatomical context. (**a**) Case 1; (**b**) Case 2; (**c**) Case 3. In each panel, the upper image is the CT slice and the lower image is the reference annotation (red, pneumothorax; blue, lung parenchyma).

**Figure 2:**
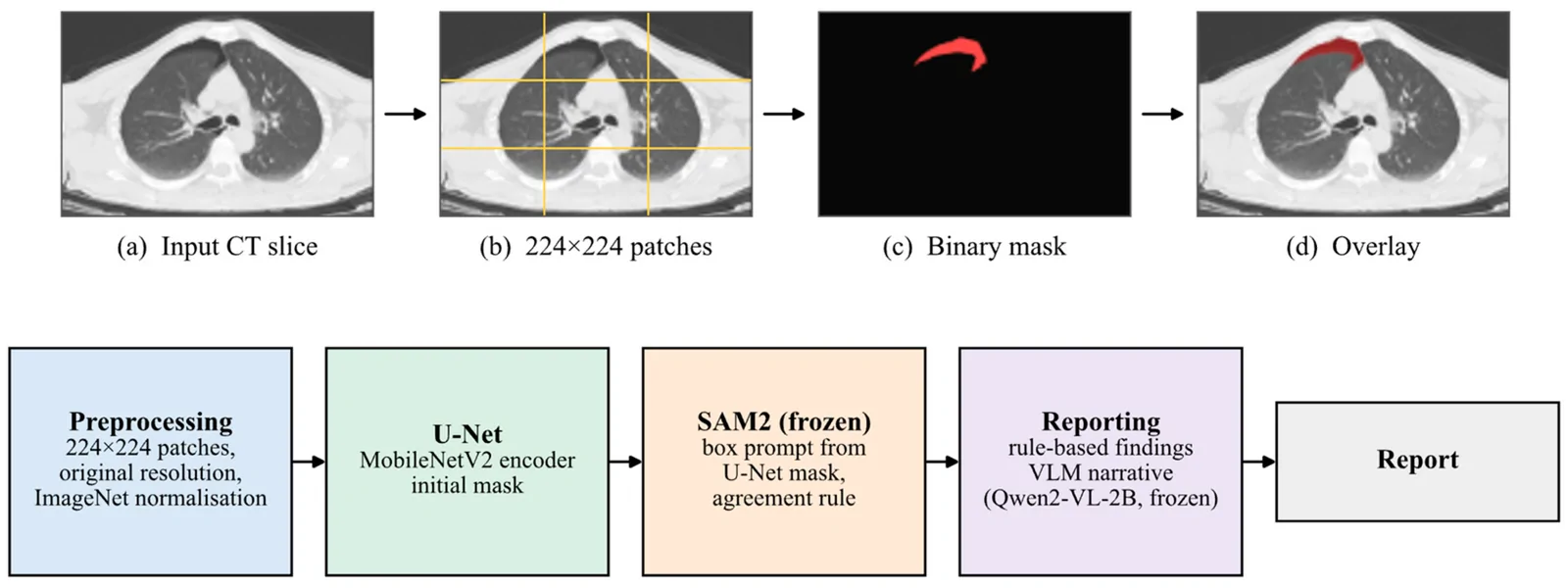
Overall architecture of the proposed SAM2- and VLM-based pneumothorax detection system. The images shown in the flow belong to an actual case from the test set (Figure 10); the binary mask and overlay were obtained from the final mask produced by the system. (**a**) Input CT slice; (**b**) division into 224×224 patches; (**c**) binary mask; (**d**) overlay of the mask on the CT slice. The lower row shows the processing modules of the system.

**Figure 3:**
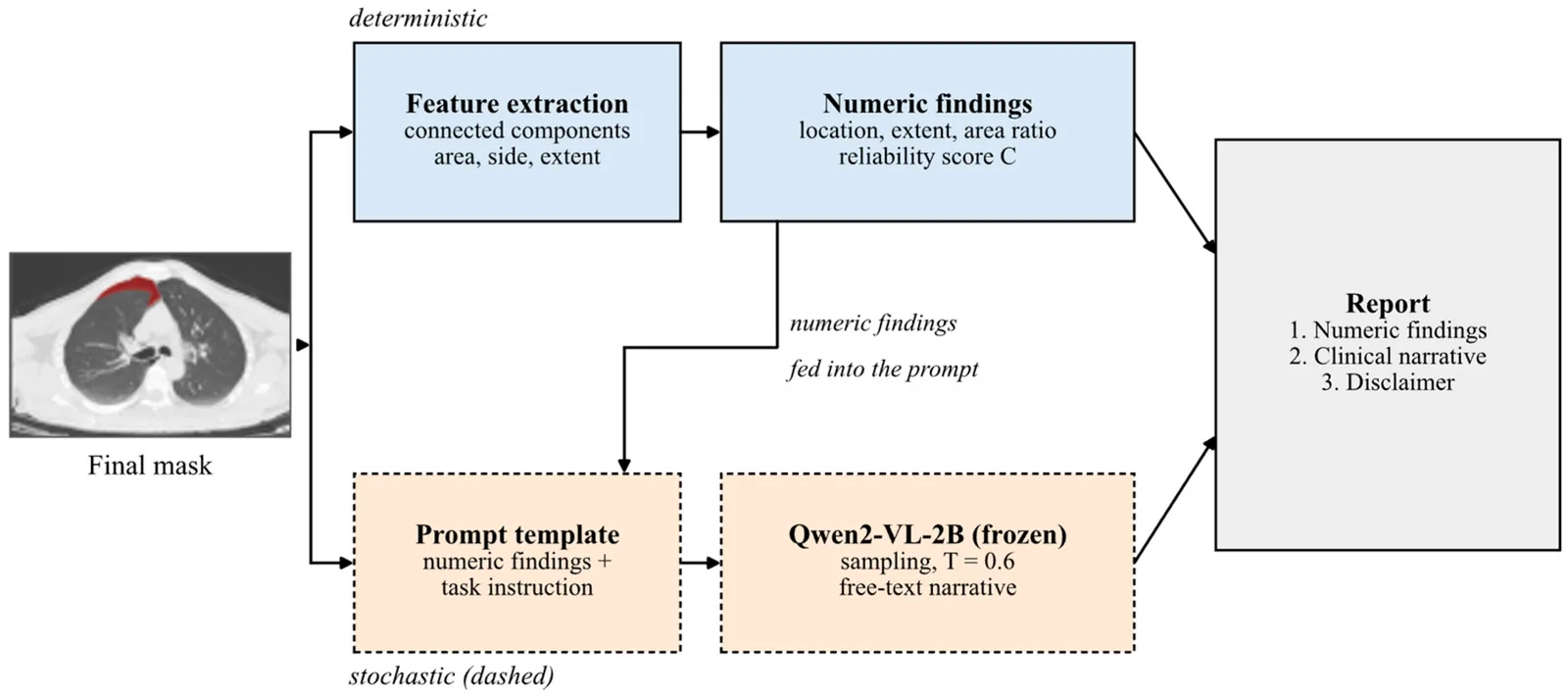
The VLM-based automatic reporting process. The overlay image used as input belongs to an actual case from the test set (Figure 10). Solid-outline boxes represent components computed in a rule-based, deterministic manner, whereas dashed-outline boxes represent stochastic components generated by sampling from a frozen vision-language model.

**Figure 4:**
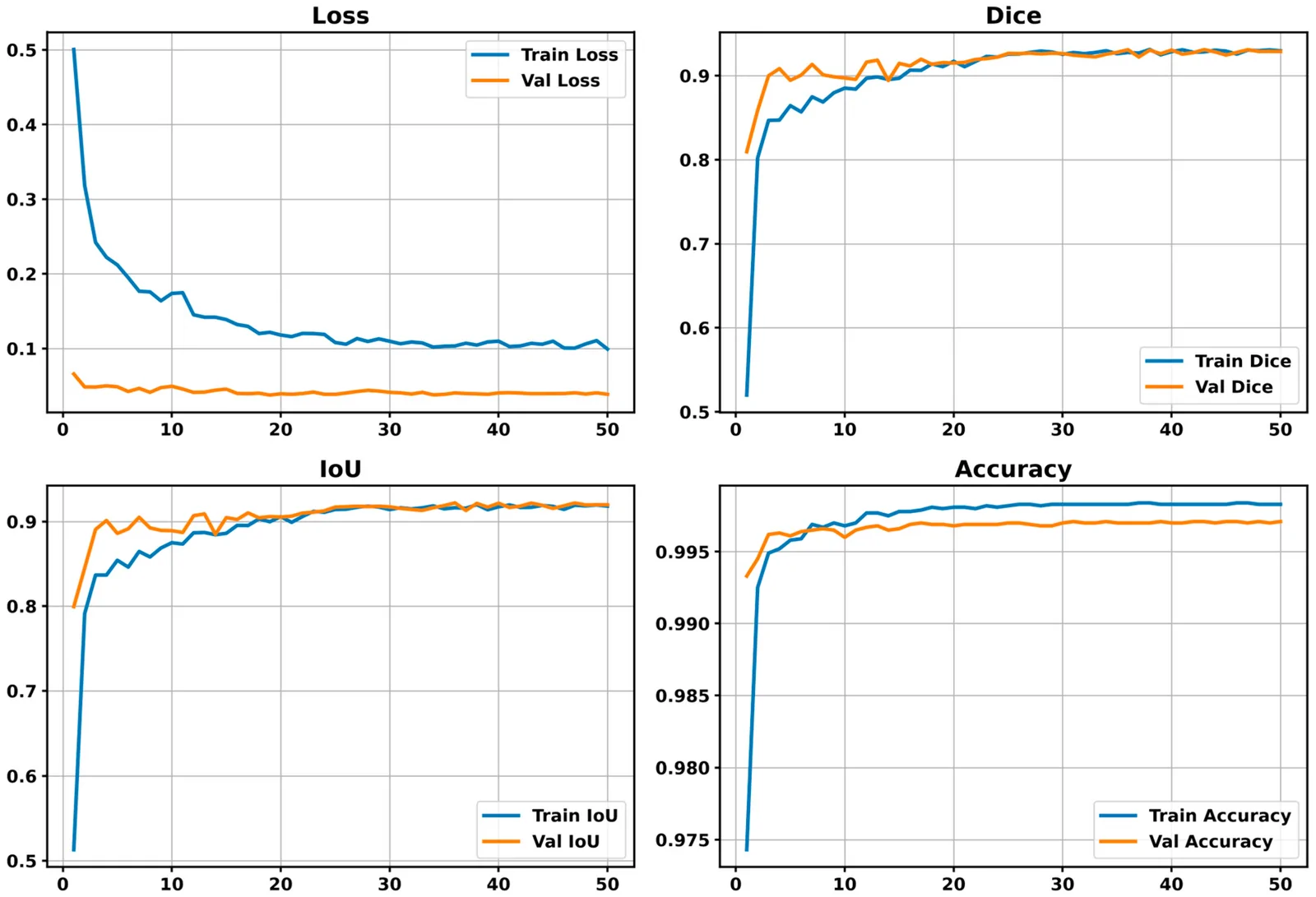
Training and validation curves: Loss; Dice coefficient; IoU and Accuracy. The dashed line indicates the epoch at which the highest validation Dice value used for model selection was obtained; the reported results correspond to this checkpoint.

**Figure 5:**
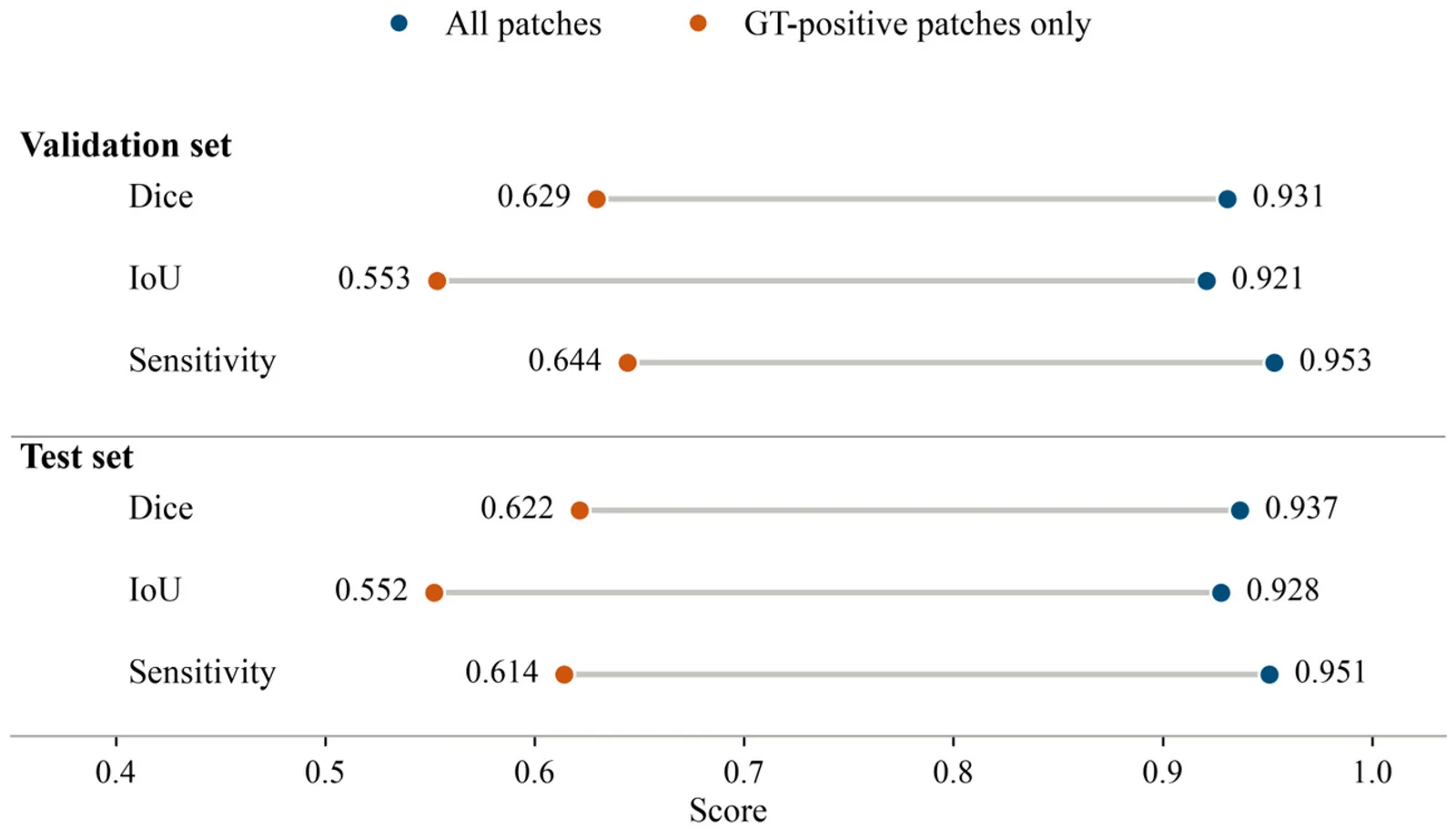
Comparison of screening performance and lesion-level segmentation quality: validation and test set. Blue points represent values computed over all patches (Table 4), orange points represent values computed only over patches with a positive ground-truth mask (Table 5); the connecting line indicates the difference between the two measurement levels.

**Figure 6:**
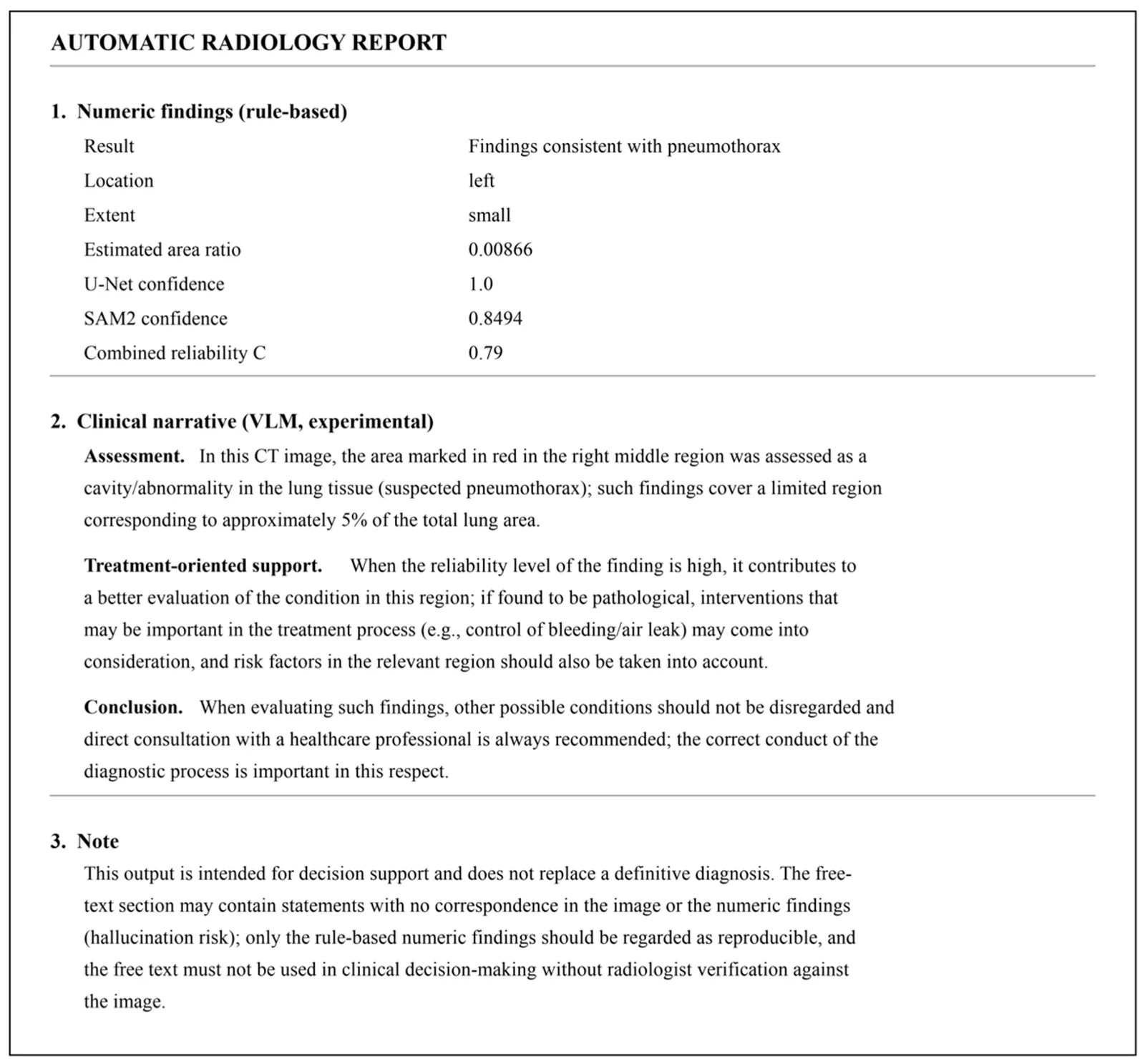
Typeset representation of the automatic report generated by the proposed system for the case shown in Figure 10. Section 1 contains the rule-based, reproducible numerical findings; Section 2 contains the free text generated by sampling from the frozen VLM (English translation of the Turkish system output); Section 3 contains the fixed disclaimer note.

**Figure 7:**
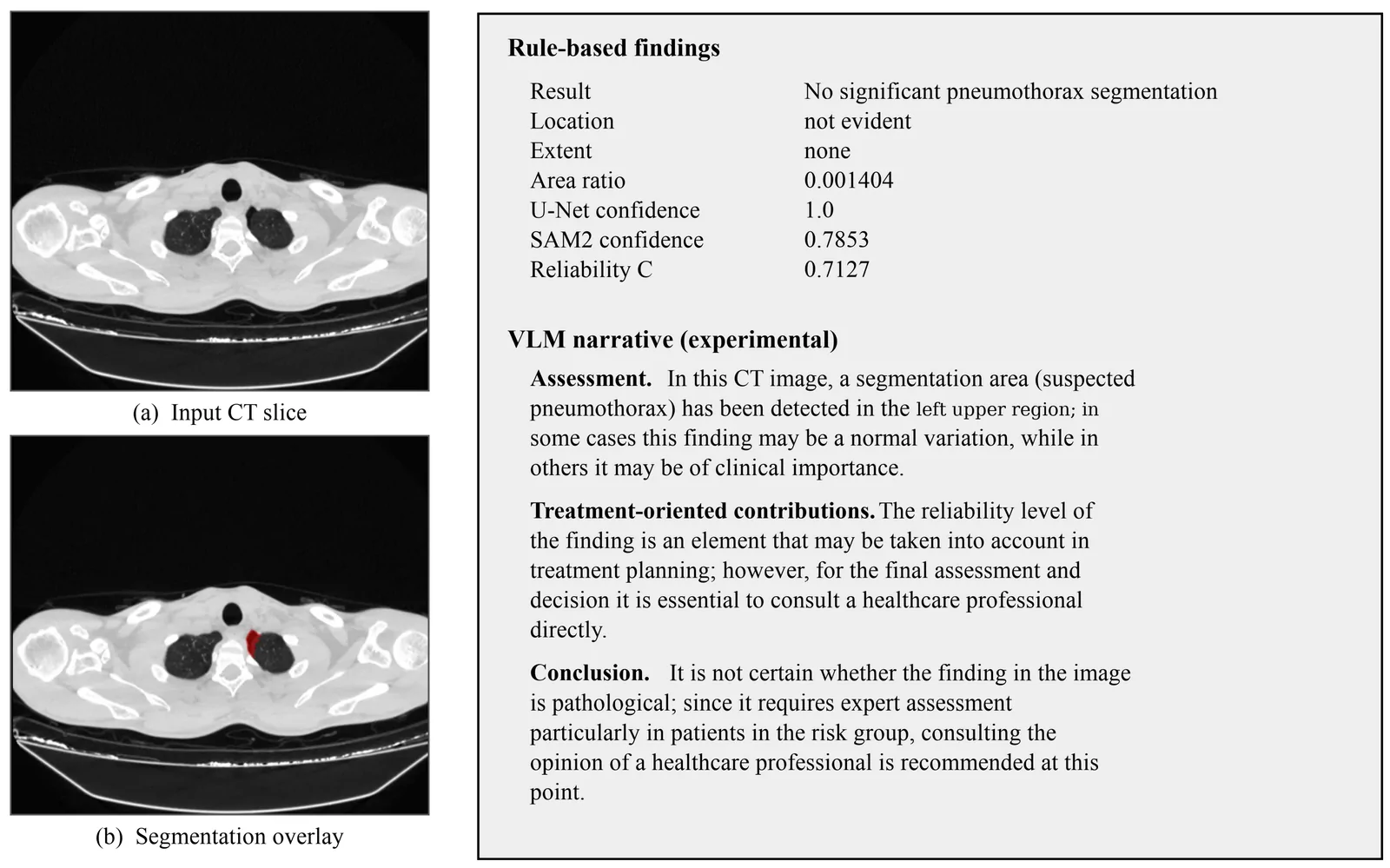
Example 1: (**a**) input CT slice; (**b**) segmentation overlay produced by the system, and the report output produced by the system. The report contains the rule-based numerical findings and the complete free-text clinical interpretation generated by the frozen VLM (English translation of the Turkish system output). Because the fixed disclaimer note at the end of each report is the same in all examples, it is shown only in Figure 6.

**Figure 8:**
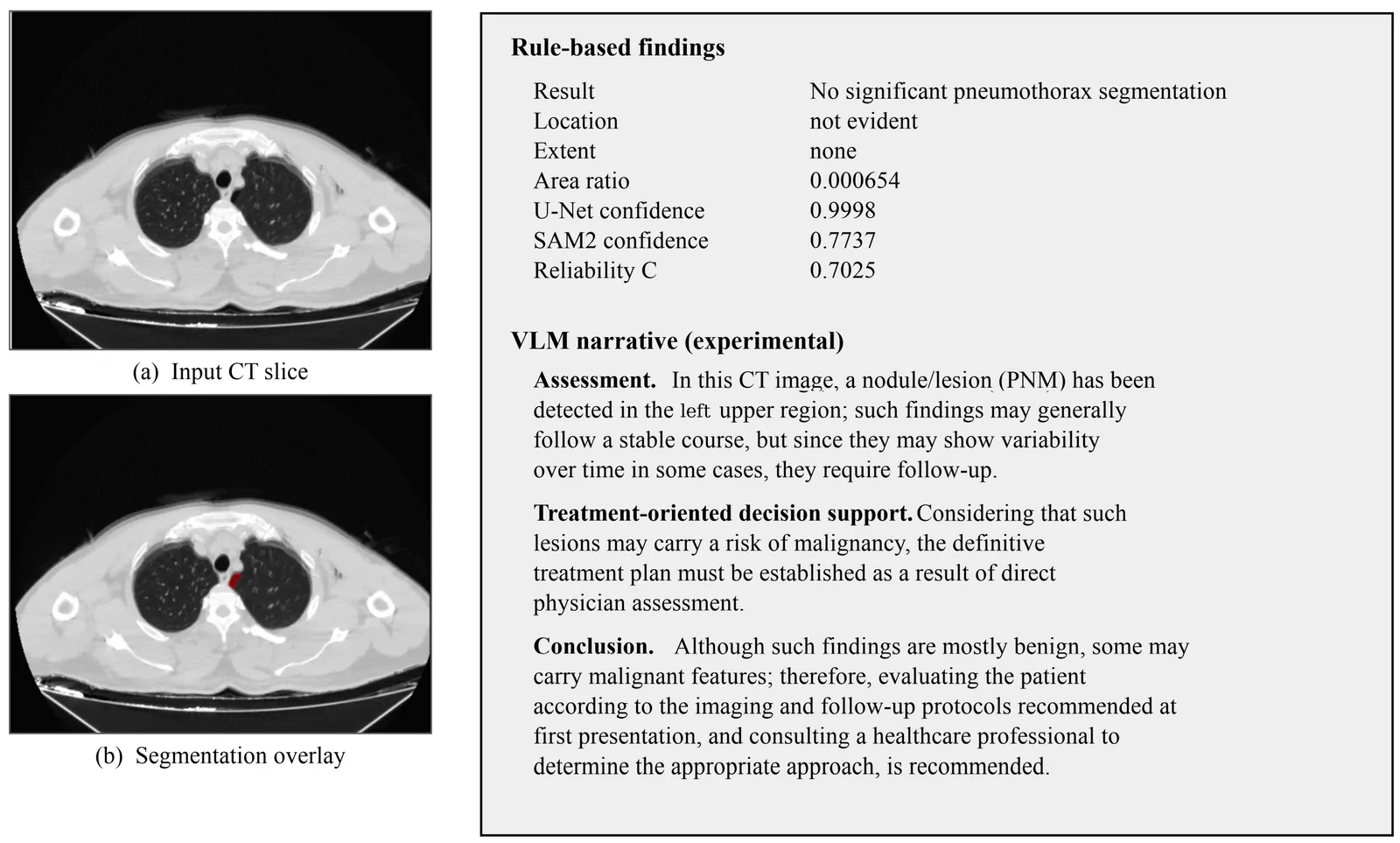
Example 2: (**a**) input CT slice; (**b**) segmentation overlay produced by the system, and the report output produced by the system. The report contains the rule-based numerical findings and the complete free-text clinical interpretation generated by the frozen VLM (English translation of the Turkish system output). Because the fixed disclaimer note at the end of each report is the same in all examples, it is shown only in Figure 6.

**Figure 9:**
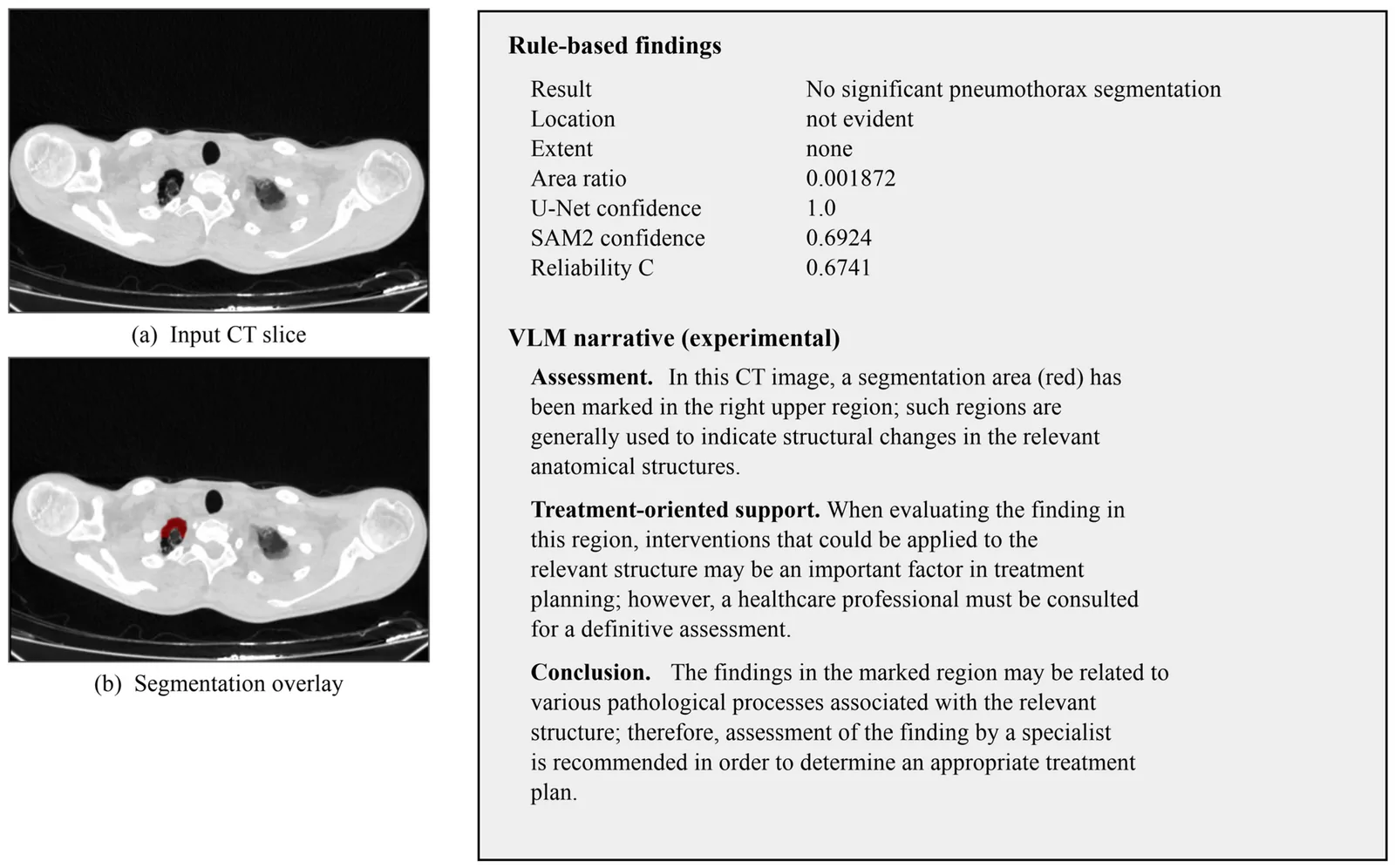
Example 3: (**a**) input CT slice; (**b**) segmentation overlay produced by the system, and the report output produced by the system. The report contains the rule-based numerical findings and the complete free-text clinical interpretation generated by the frozen VLM (English translation of the Turkish system output). Because the fixed disclaimer note at the end of each report is the same in all examples, it is shown only in Figure 6.

**Figure 10:**
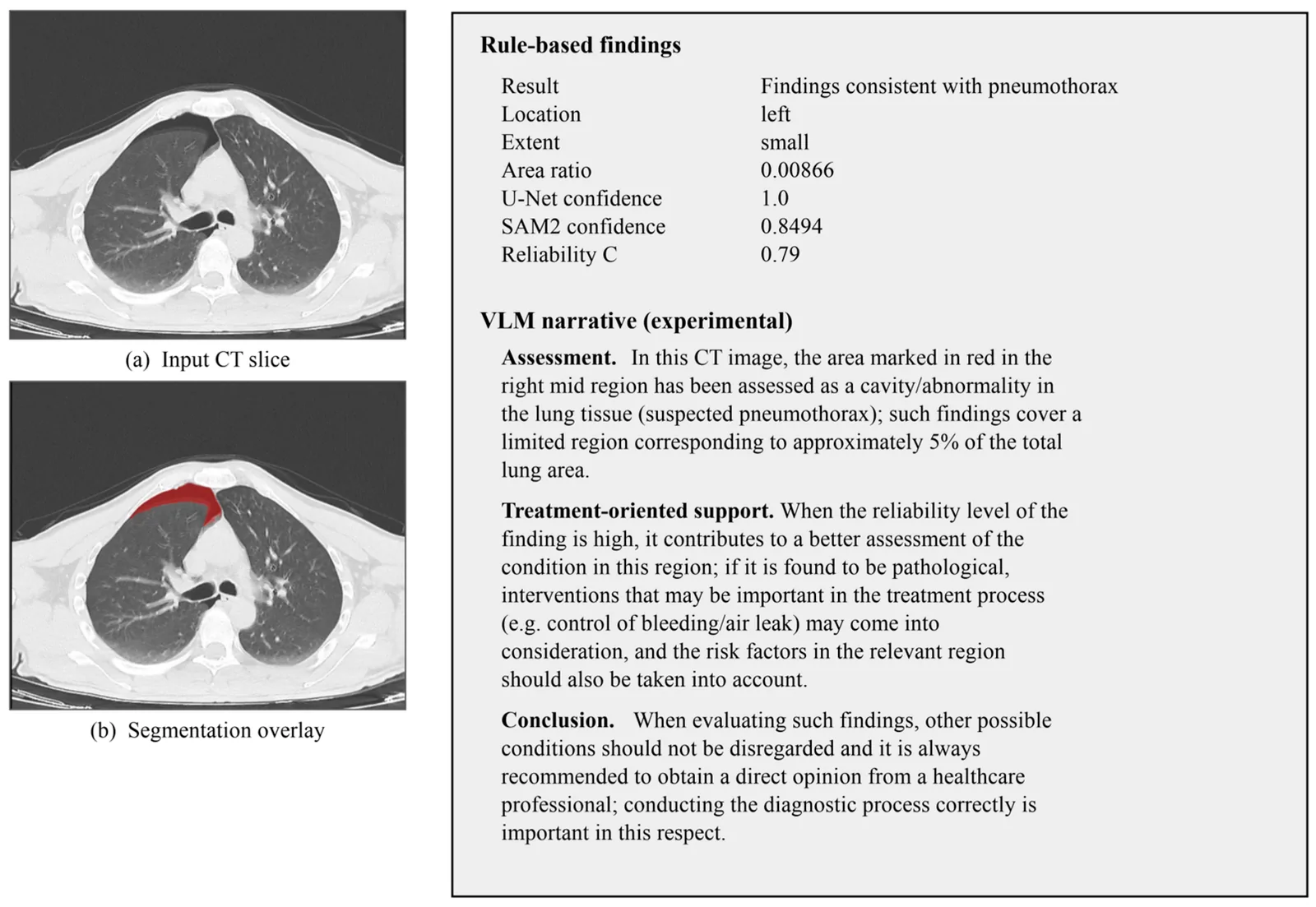
Example 4: (**a**) input CT slice; (**b**) segmentation overlay produced by the system, and the report output produced by the system. The report contains the rule-based numerical findings and the complete free-text clinical interpretation generated by the frozen VLM (English translation of the Turkish system output). Because the fixed disclaimer note at the end of each report is the same in all examples, it is shown only in Figure 6.

**Figure 11:**
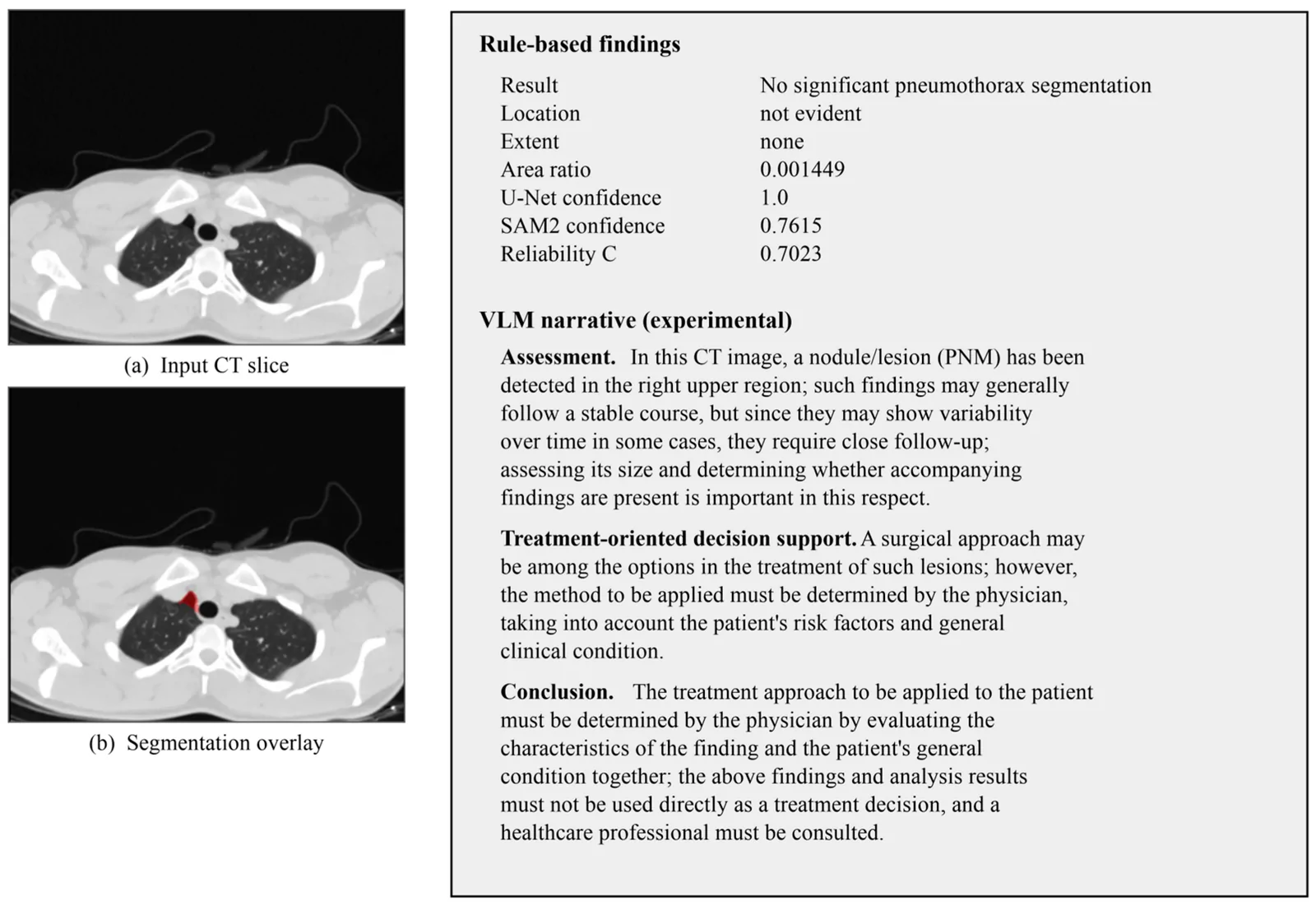
Example 5: (**a**) input CT slice; (**b**) segmentation overlay produced by the system, and the report output produced by the system. The report contains the rule-based numerical findings and the complete free-text clinical interpretation generated by the frozen VLM (English translation of the Turkish system output). Because the fixed disclaimer note at the end of each report is the same in all examples, it is shown only in Figure 6.

**Figure 12:**
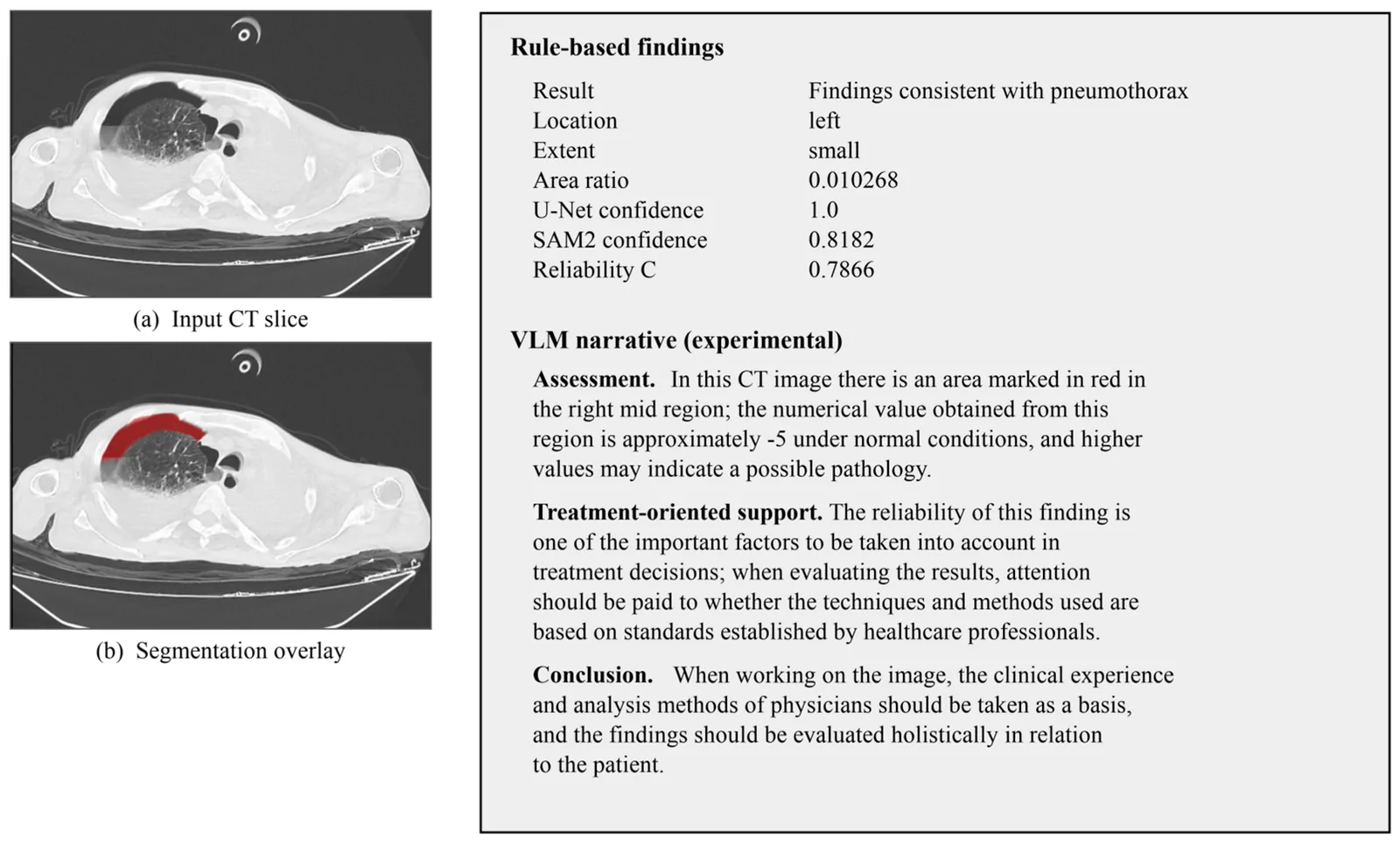
Example 6: (**a**) input CT slice; (**b**) segmentation overlay produced by the system, and the report output produced by the system. The report contains the rule-based numerical findings and the complete free-text clinical interpretation generated by the frozen VLM (English translation of the Turkish system output). Because the fixed disclaimer note at the end of each report is the same in all examples, it is shown only in Figure 6.

**Table 1:** Summary of representative literature on pneumothorax/chest segmentation and automatic radiological reporting methods.

Study	Modality	Method	Reported Result	Key Gap Addressed by This Study
Ronneberger et al. [13]	Biomedical images (general)	U-Net (encoder–decoder, skip connections)	Foundational segmentation architecture	Segmentation only; no boundary refinement or reporting stage
Abedalla et al. [29]	Chest radiography	U-Net with EfficientNet/ResNet encoder ensembles	Dice 0.8124	Segmentation only; single modality (CXR); no automatic clinical reporting
Sae-Lim et al. [30]	Chest radiography	Two-stage deep learning segmentation + quantification	Dice 0.8860	Quantification only; no free-text reporting or confidence estimation
Kirillov et al. [19]; Ravi et al. [20]	Natural images/video	SAM/SAM2 prompted foundation segmentation	Strong zero-shot generalization on natural images	Not validated for CT pneumothorax boundary delineation with frozen weights
Zhu et al. [21]	Medical images (video-based)	Medical SAM2	Strong performance on selected medical benchmarks	Requires domain-specific fine-tuning; not coupled to a reporting module
Jing et al. [33]	Chest radiography	CNN-RNN automatic report generation	Descriptive report generation	No explicit geometric grounding of text in a segmentation mask; not evaluated for pneumothorax
Boecking et al. [34]	Chest radiography	Contrastive biomedical vision-language pretraining	Improved report-retrieval performance	Pretraining framework; not an end-to-end detection-to-report pipeline
Li et al. [37]	Multiple medical modalities (survey)	Review of VLM taxonomy, from fusion to general large models	Synthesis of >100 studies	Identifies the scarcity of segmentation-grounded VLM reporting pipelines, motivating this study
Proposed method	Chest CT	U-Net + frozen SAM2 refinement + frozen VLM reporting	Dice 0.9368 (all patches)/0.6215 (lesion-level), test set	Combines segmentation, boundary refinement, and structured + free-text reporting with an explicit confidence coefficient in one pipeline

**Table 2:** Distribution of the dataset at the patient and patch level.

Subset	Number of Patches	Number of Patients	Positive Patches	Purpose
Training	9939	111	1383	Model training
Validation	1625	17	214	Model selection and learning-rate scheduling
Test	3991	43	508	Performance evaluation
Total	15,555	171	2105	

**Table 3:** U-Net training parameters.

Parameter	Value
Encoder	MobileNetV2 (ImageNet-pretrained)
Number of epochs	50
Batch size	16
Learning rate	0.0001
Learning-rate schedule	ReduceLROnPlateau (factor 0.5, patience 2 epochs)
Optimization algorithm	Adam [22]
Loss function	0.5 × Dice + 0.5 × Binary Cross-Entropy (BCE)
Hardware (GPU)	NVIDIA Tesla T4

**Table 4:** Performance results of the U-Net initial segmentation and the SAM2 refinement (HD95 is in pixel units and was calculated only over samples in which both the ground-truth and predicted masks were non-empty; a random subset of *n* ≈ 300 samples was used for the U-Net + SAM2 columns. In binary segmentation, the F1 metric is mathematically identical to Dice; the values given in the table have been corrected according to this identity. Values were computed per sample and then averaged; since Dice, Sensitivity, and Accuracy take the value 1.0 by definition for patches with an empty ground-truth mask, and approximately 86.5% of the patches fall into this group, these rows predominantly reflect screening performance. For lesion-level values, see Table 5.)

Metric	U-Net (Validation)	U-Net (Test)	U-Net + SAM2 (Validation)	U-Net + SAM2 (Test)
Loss	0.0363	–	–	–
Dice	0.9309	0.9368	0.9325	0.9244
IoU	0.9209	0.9279	0.9234	0.9129
Accuracy	0.9971	0.9979	0.9946	0.9977
Sensitivity	0.9532	0.9509	0.9572	0.9427
Specificity	0.9980	0.9990	0.9947	0.9984
F1	0.9309	0.9368	0.9325	0.9244
HD95	34.81	27.53	51.84	29.36

**Table 5:** U-Net segmentation quality and patch-level screening performance on patches with a positive ground-truth (GT) mask only (the HD95 value is defined only for samples in which both the ground-truth and predicted masks are non-empty, and is therefore identical to the U-Net value in Table 4; it is not a separate calculation. Patch-level screening sensitivity and specificity indicate the per-patch accuracy of the decision as to whether a patch contains pneumothorax.)

Metric (GT-Positive Patches Only)	Validation	Test
Dice	0.6295	0.6215
IoU	0.5534	0.5519
Sensitivity	0.6443	0.6142
Specificity	0.9889	0.9942
HD95 (pixels)	34.81	27.53
*n* (positive patches/negative patches)	214/1411	508/3483
Patch-level screening sensitivity	0.8224	0.7992
Patch-level screening specificity	0.9766	0.9828

**Table 6:** Comparison with pneumothorax segmentation studies in the literature.

Study	Modality	Method	Dice/IoU
Abedalla et al. [29]	Chest radiography	U-Net (EfficientNet + ResNet)	0.8124/0.7245
Sae-Lim et al. [30]	Chest radiography	Two-stage deep learning	0.8860/-
Proposed method	CT	U-Net (alone)	0.9368/0.9279 (test)
Proposed method	CT	U-Net + SAM2 (full pipeline)	0.9244/0.9129 (test subsample, *n* ≈ 300)
Proposed method (lesion-level)	CT	U-Net (GT-positive patches only)	0.6215/0.5519 (test)

## Data Availability

The dataset used in this study is not publicly available due to patient privacy and can be obtained from the corresponding author upon reasonable request.

## Abbreviations

The following abbreviations are used in this manuscript:

CT	Computed Tomography
SAM2	Segment Anything Model 2
VLM	Vision-Language Model
IoU	Intersection over Union
HD95	95th-Percentile Hausdorff Distance
BLEU	Bilingual Evaluation Understudy
ROUGE	Recall-Oriented Understudy for Gisting Evaluation
CNN	Convolutional Neural Network
ViT	Vision Transformer
SHAP	SHapley Additive exPlanations
Grad-CAM	Gradient-weighted Class Activation Mapping
GPU	Graphics Processing Unit
BCE	Binary Cross-Entropy
DICOM	Digital Imaging and Communications in Medicine

## Appendix A

Table A1, presented below, shows the main layer blocks, output tensor dimensions, and trainable-parameter distribution of the U-Net architecture with a MobileNetV2-based encoder.

**Table A1:** Layer/parameter breakdown of the U-Net architecture (summarized at the encoder–decoder level).

Layer Block/Type	Input Size	Output Size	Trainable Parameters
Input Image	(224, 224, 3)	(224, 224, 3)	0
Encoder (MobileNetV2, ImageNet-pretrained)	(224, 224, 3)	(7, 7, 1280)	2,223,872
Decoder (U-Net Decoder, 5 upsampling blocks)	(7, 7, 1280)	(224, 224, 16)	4,404,928
Segmentation Head (3×3 Conv2D)	(224, 224, 16)	(224, 224, 1)	145
TOTAL	—	—	6,628,945

## Appendix B. VLM Prompt Template

The structured prompt used to guide the frozen VLM (Section 2.5) is given below in English translation; the deployed system itself operates in Turkish. The prompt combines a fixed instruction template with the case-specific rule-based numerical fields (location, extent, area ratio, and the three confidence scores), which are substituted into the placeholders shown in braces before the prompt is sent to the model together with the segmentation overlay image.

“You are a chest-radiology decision-support assistant. Below is a lung CT/PA image and a pneumothorax segmentation overlay obtained with U-Net + SAM2. The red-marked region shows the area the model has marked as pneumothorax. Numerical findings: Location: {location}; Extent: {extent}; Estimated area ratio: {area_ratio}; U-Net confidence score: {unet_confidence}; SAM2 confidence score: {sam2_confidence}; Combined reliability coefficient: {combined_confidence}. Your task: based on this information and the image, write three paragraphs of complete sentences in Turkish. Each paragraph must begin with one of the following headings, with at least 2–3 sentences of genuine clinical commentary beneath it: (1) Assessment: interpret the image findings and the numerical values above. (2) Treatment-Related Decision Support: suggest possible next clinical steps according to the reliability level. (3) Conclusion: give a brief summary of the findings. Important: write only these three paragraphs; do not repeat this instruction text, the heading explanations, or the following sentence verbatim. Instead, state in your own words, naturally, that this output is not a definitive diagnosis and is a decision-support output.”
